# Supplementary material for: Looking for the sponge loop: analyses of detritus on a Caribbean forereef using stable isotope and eDNA metabarcoding techniques
Source: PeerJ. 2024 Feb 23;12:e16970. doi: 10.7717/peerj.16970 (PMC10896084; doi:10.7717/peerj.16970)
Supplement: Supplemental Information 1 [file peerj-12-16970-s001.docx]

***The following supplements accompany the article:***

Looking for the sponge loop: Analyses of detritus on a Caribbean forereef using stable isotope and eDNA metabarcoding techniques

Lauren K. Olinger^1,2^*, Beverly McClenaghan^3^, Mehrdad Hajibabaei^3,4^, Nicole Fahner^3^, Lesley Berghuis^3^, Hoda Rajabi^3^, Patrick Erwin^1^, Chad S. Lane^5^, Joseph R. Pawlik^1^

^1^ Department of Biology and Marine Biology, University of North Carolina Wilmington, Wilmington, NC, USA

^2^ Center for Marine and Environmental Studies, University of the Virgin Islands, Saint Thomas, VI, USA

^3^ Centre for Environmental Genomics Applications, eDNAtec Inc., Newfoundland and Labrador, St. John's, Canada

^4^ Department of Integrative Biology, University of Guelph, Guelph, ON, Canada

^5^ Department of Earth and Ocean Sciences, University of North Carolina Wilmington, Wilmington, NC, USA

* Corresponding author: [l.olinger12993@gmail.com](mailto:l.olinger12993@gmail.com)

# Environmental DNA Sample Laboratory Processing

## DNA Extraction

DNA extraction protocols were tested for each sample type and optimized to maximize the DNA yield from the samples given their small size (<0.25 g). The optimized protocols are described below.

For sponge tissue, a subsample of up to 0.025 g was collected. The tube was left open under a laminar flow hood for 5 mins to allow excess ethanol to evaporate. DNA was extracted from sub-samples using the DNeasy Blood and Tissue Kit (Qiagen, Hilden, Germany) following the manufacturer’s protocol.

For detritus samples, DNA was extracted from detritus samples using the DNeasy PowerSoil Kit (Qiagen). Samples were centrifuged, and ethanol was removed, then ~ 200uL of the lysis buffer from the bead tube was added to the sediment, mixed, and then transferred back to the bead tube. Extraction was then completed following the manufacturer’s protocol with the additional of two steps. After the addition of C1 and homogenization, 25 µL of ProK was added and the samples were vortexed before centrifugation. The manufacturer’s protocol was then followed until the elution step, where warmed C6 buffer was passed through the spin column twice to elute the DNA.

DNA was extracted from feces samples and from algae and cyanobacteria tissue using the DNeasy PowerSoil Kit (Qiagen). Sample material was transferred into bead tubes and the extraction was completed following the manufacturer’s protocol with the exception of three additional steps. After the addition of C1 and homogenization, 25 µL of ProK was added and samples were vortexed for 40 seconds. Samples were then incubated at 65°C for one hour and held at 37°C overnight before centrifugation. The manufacturer’s protocol was then followed until the elution step, where warmed C6 buffer was passed through the spin column twice to elute the DNA.

All DNA extracts were quantified using the Quant-iT PicoGreen dsDNA assay with a Synergy HTX plate fluorometer (BioTek, Winooski, VT, USA).

## Library Preparation

Three DNA markers from three gene regions (cytochrome c oxidase I (COI), 16S rRNA and 18S rRNA) were amplified from each marker using PCR to assess metazoan, algal, and cyanobacteria diversity (ESM Tables 1-2). Each PCR reaction contained 1X reaction buffer, 2 mM MgCl_2_, 0.2mM dNTPs, 0.2 μM of each of the forward and reverse Illumina-tailed primers, 1.5U Platinum Taq (Invitrogen, Carlsbad, CA, USA) and 1.2 μL of DNA in a total volume of 15 μL. See ESM Table 2 for PCR conditions for all primer sets. Three PCR replicates were performed for each primer set from each sample and then pooled for a single PCR cleanup with the QIAquick 96 PCR purification kit (Qiagen).

Amplicons were visualized using agarose gel (1.5% w/v) electrophoresis to verify amplification of DNA markers and to assess negative controls generated during PCR and extraction. Negative controls were carried through to sequencing as an added level of verification. Amplicons were then indexed using unique dual Nextera indexes (IDT, Coralville, IA, USA; 8-bp index codes). Indexing PCR conditions were initiated for 3 mins at 95°C, followed by 12 cycles of 95°C for 30 s, 55°C for 30 s, and 72°C for 30 s, and a final extension at 72°C for 5 mins. Amplicons were quantified with Quant-iT PicoGreen dsDNA assay and pooled together in equimolar concentrations by DNA marker. Amplicon pools were cleaned using AMPure XP cleanups, quantified with a Qubit fluorometer (Thermo Fisher, Waltham, MA, USA) and the size distribution of each pool was verified with the DNA 7500 kit on the Agilent 2100 Bioanalyzer. The amplicon pools were combined into one sequencing library. The library was sequenced with a 500-cycle SP kit on the Illumina NovaSeq 6000 following the NovaSeq standard workflow with a target minimum sequencing depth of 250,000 sequences per sample per amplicon***.***
