# Supplementary figures and images for: Looking for the sponge loop: analyses of detritus on a Caribbean forereef using stable isotope and eDNA metabarcoding techniques
Source: PeerJ. 2024 Feb 23;12:e16970. doi: 10.7717/peerj.16970 (PMC10896084; doi:10.7717/peerj.16970)

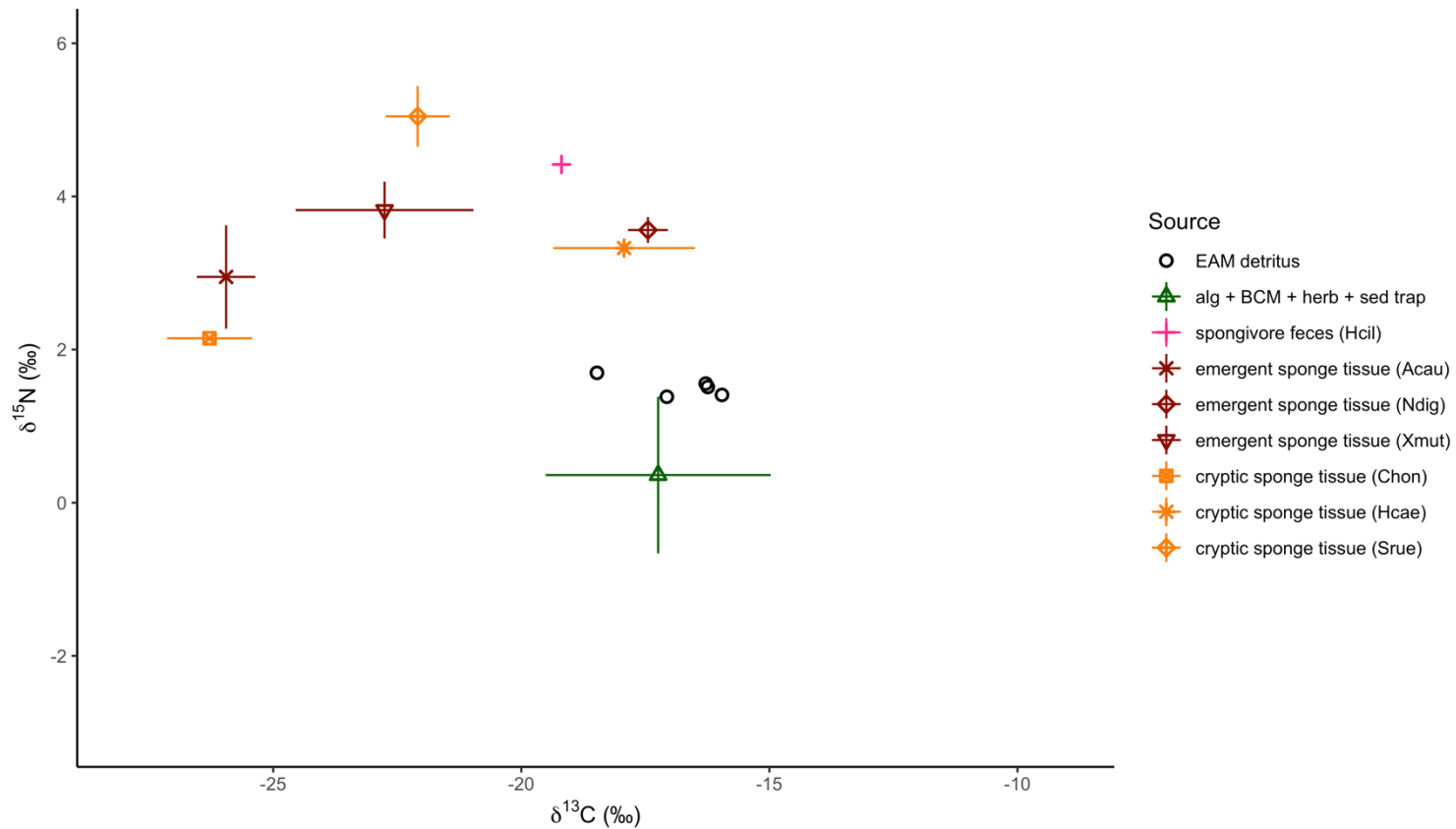

Supplement: Figure S1 — EAM detritus samples are represented by open circles, respectively, that denote the values of individual samples. All other symbols represent the average ± SD of source samples denoted in legend. [file peerj-12-16970-s002.pdf]

Source

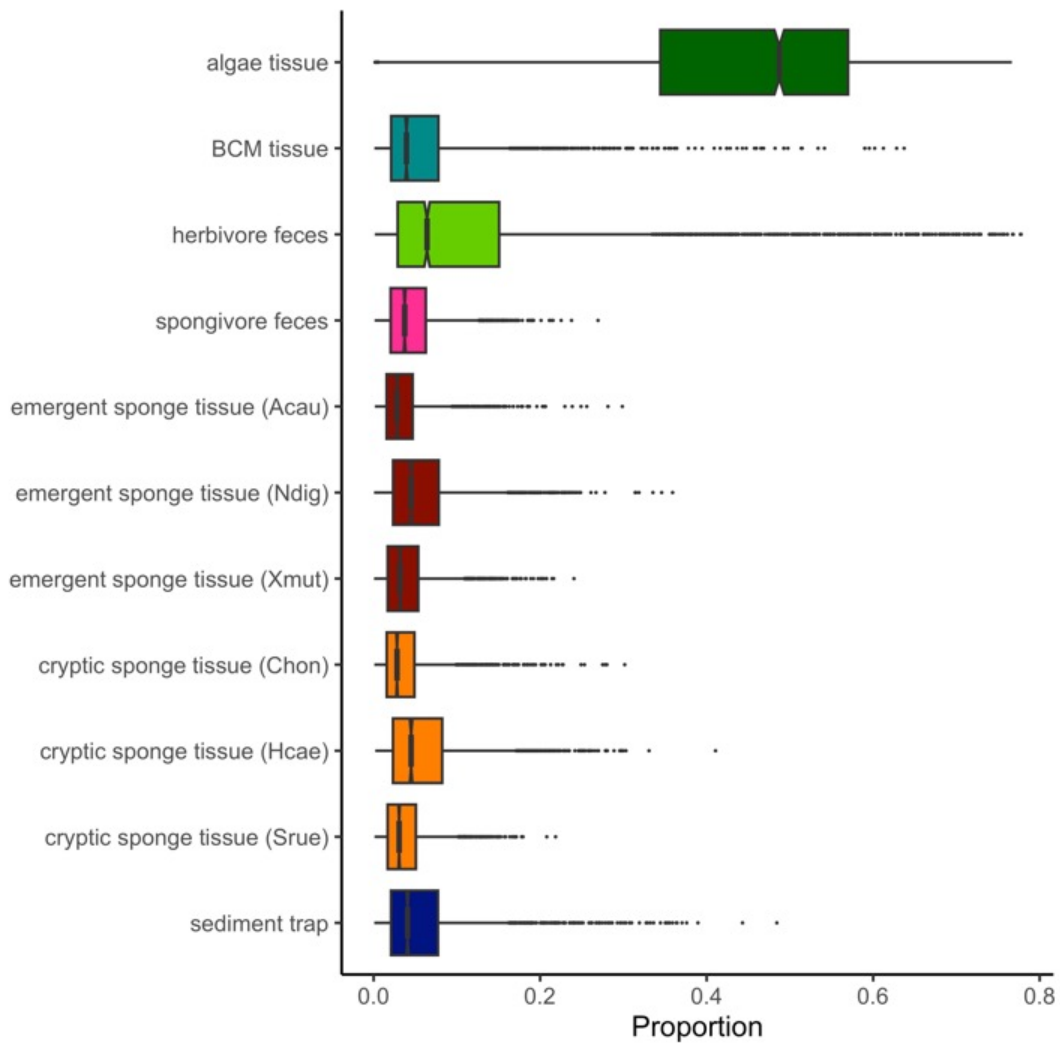

Supplement: Figure S2 — In the box plots, the boundary of the box closest to zero indicates the 25th percentile, a black line within the box marks the median, and the boundary of the box farthest from zero indicates the 75th percentile. Whiskers above and below the box indicate the 10th and 90th percentiles. Points above and below the whiskers indicate outliers outside the 10th and 90th percentiles. [file peerj-12-16970-s003.pdf]

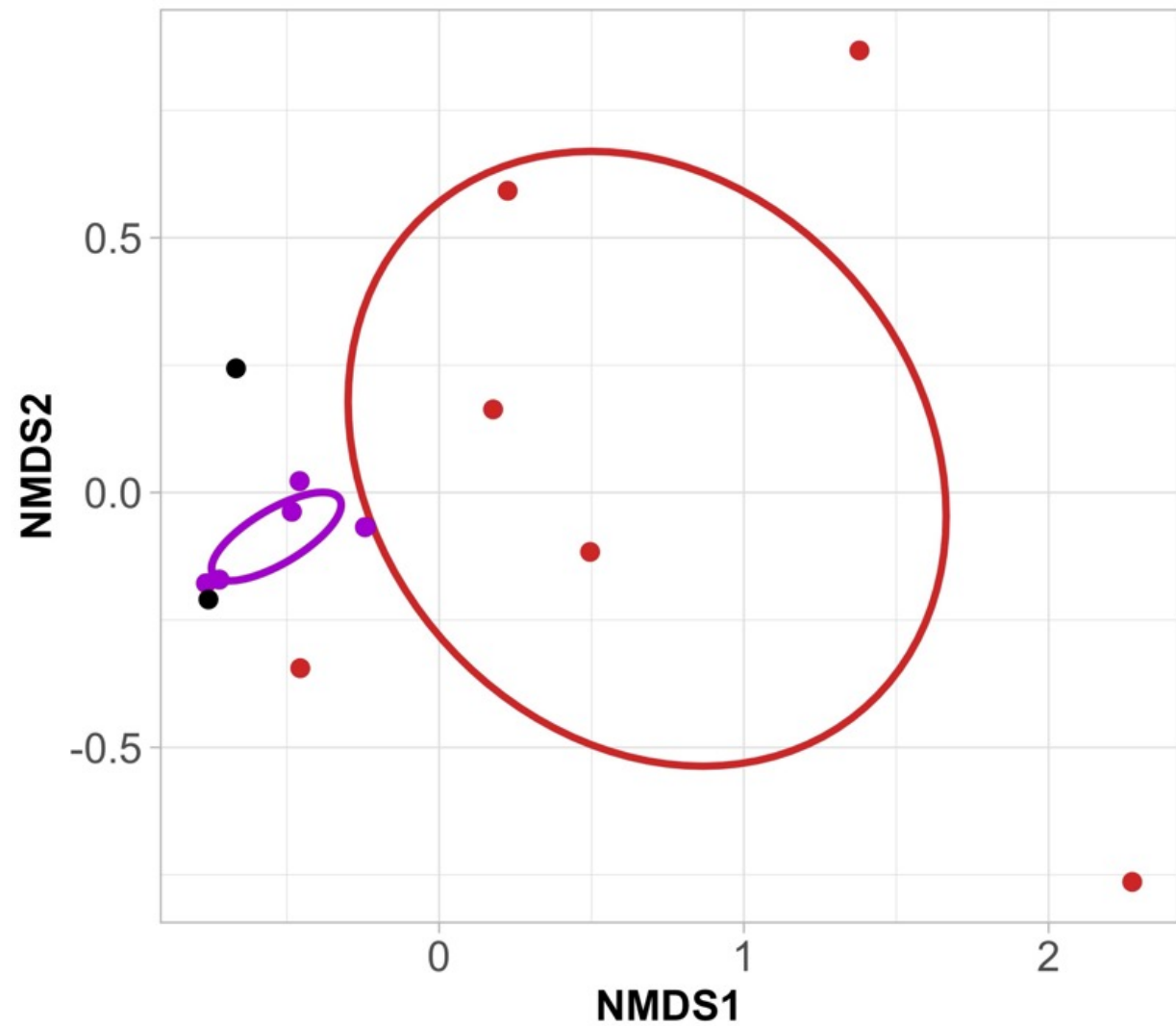

2018 EAM detritus 2019 EAM detritus tray detritus

Supplement: Figure S3 — Points represent individual samples, and 95% confidence ellipses indicate sampling distributions for each detritus type. [file peerj-12-16970-s004.pdf]
