# Supplementary material for: Looking for the sponge loop: analyses of detritus on a Caribbean forereef using stable isotope and eDNA metabarcoding techniques
Source: PeerJ. 2024 Feb 23;12:e16970. doi: 10.7717/peerj.16970 (PMC10896084; doi:10.7717/peerj.16970)
Supplement: Table S1 [file peerj-12-16970-s005.docx]

| **Primer Name** | **Gene Region** | **Forward Primer** | **Reverse Primer** | **Source** |
| --- | --- | --- | --- | --- |
| F230 | COI | 5'-GGTCAACAAATCATAAAGATATTGG-3' | 5'-CTTATRTTRTTTATNCGNGGRAANGC-3' | 1 |
| 18SV4M1 | 18S rRNA | 5'-GCAGTTAAAAAGCTCGTAG-3' | 5'-TCCAAGAATTRCACCTCT-3' | 2 |
| 16S27F534R | 16S rRNA | 5'-AGAGTTTGATCCTGGCTCAG-3' | 5'-ATTACCGCGGCTGCTGG-3' | 3-4 |
| ^1^Gibson JF, Shokralla S, Curry C, Baird DJ, Monk WA, King I, Hajibabaei M (2015) Large-scale biomonitoring of remote and threatened ecosystems via high-throughput sequencing. PLOS ONE 10:e0138432  ^2^Stat M, Huggett MJ, Bernasconi R, DiBattista JD, Berry TE, Newman SJ, Harvey ES, Bunce M (2017) Ecosystem biomonitoring with eDNA: metabarcoding across the tree of life in a tropical marine environment. Sci Rep 7:12240  ^3^Muyzer G, de Waal EC, Uitterlinden AG (1993) Profiling of complex microbial populations by denaturing gradient gel electrophoresis analysis of polymerase chain reaction-amplified genes coding for 16S rRNA. Applied and Environmental Microbiology 59:695–700  ^4^Weisburg WG, Barns SM, Pelletier DA, Lane DJ (1991) 16S ribosomal DNA amplification for phylogenetic study. Journal of Bacteriology 173:697–703 | | | | |
