# Supplementary material for: Looking for the sponge loop: analyses of detritus on a Caribbean forereef using stable isotope and eDNA metabarcoding techniques
Source: PeerJ. 2024 Feb 23;12:e16970. doi: 10.7717/peerj.16970 (PMC10896084; doi:10.7717/peerj.16970)
Supplement: Supplemental Information 6 [file peerj-12-16970-s006.docx]

| **DNA Marker** | **Amplicon Size** | **Initial** | **# Cycles** | **Denaturation** | **Annealing** | **Extension** | **Final Extension** |
| --- | --- | --- | --- | --- | --- | --- | --- |
| F230 | 226-235 | 3 min 95°C | 35 | 30 sec 94°C | 40 sec 46°C | 60 sec 72°C | 10 min 72°C |
| 18SV4M1 | 309 | 3 min 95°C | 35 | 30 sec 94°C | 30 sec 46°C | 30 sec 72°C | 10 min 72°C |
| 16S | 507 | 3 min 94°C | 35 | 45 sec 94°C | 45 sec 48°C | 90 sec 72°C | 30 sec 72°C |
