# Supplementary material for: Looking for the sponge loop: analyses of detritus on a Caribbean forereef using stable isotope and eDNA metabarcoding techniques
Source: PeerJ. 2024 Feb 23;12:e16970. doi: 10.7717/peerj.16970 (PMC10896084; doi:10.7717/peerj.16970)
Supplement: Table S3 — Composite samples are indicated in bold. [file peerj-12-16970-s007.docx]

|  |  |  | July 2018 | | | | | March 2019 | | | | |
| --- | --- | --- | --- | --- | --- | --- | --- | --- | --- | --- | --- | --- |
| category | sample type | species | n | ave | sd | min | max | n | ave | sd | min | max |
| detritus | **EAM** | total | 5 | -16.8 | 1.0 | -18.5 | -16.0 | 11 | -17.6 | 1.2 | -19.5 | -15.1 |
|  | **tray** | total | 0 | - | - | - | - | 3 | -17.0 | 2.3 | -18.9 | -14.4 |
| source | algae tissue | *Dictyota sp.* | 5 | -15.5 | 0.9 | -16.2 | -14.1 | 5 | -16.8 | 0.9 | -18.1 | -15.9 |
|  |  | *Halimeda sp.* | 0 | - | - | - | - | 3 | -15.1 | 3.2 | -18.8 | -13.0 |
|  |  | *Lobophora variegata* | 5 | -14.6 | 0.7 | -15.7 | -14.0 | 6 | -14.8 | 1.4 | -17.4 | -13.5 |
|  |  | total | 10 | -15.1 | 0.9 | -16.2 | -14.0 | 14 | -15.6 | 1.9 | -18.8 | -13.0 |
|  | BCM tissue | total | 3 | -21.0 | 0.1 | -21.2 | -21.0 | 5 | -27.1 | 0.5 | -27.7 | -26.5 |
|  | **herbivore feces** | *Acanthurus bahianus* | 3 | -17.9 | 1.1 | -19.0 | -16.9 | 4 | -18.4 | 1.1 | -19.8 | -17.3 |
|  |  | *Acanthurus coeruleus* | 2 | -17.9 | 0.2 | -18.0 | -17.7 | 1 | -19.7 | - | - | - |
|  |  | total | 5 | -17.9 | 0.8 | -19.0 | -16.9 | 5 | -18.7 | 1.1 | -19.8 | -17.3 |
|  | **spongivore feces** | *Holacanthus ciliaris* | 1 | -19.2 | - | - | - | 1 | -18.7 | - | - | - |
|  |  | *Pomacanthus paru* | 0 | - | - | - | - | 2 | -20.8 | 0.0 | -20.8 | -20.8 |
|  |  | total | 1 | -19.2 | - | - | - | 3 | -20.1 | 1.2 | -20.8 | -18.7 |
|  | emergent sponge tissue | *Aplysina cauliformis* | 10 | -25.9 | 0.6 | -27.2 | -25.3 | 10 | -17.4 | 1.2 | -19.5 | -16.1 |
|  |  | *Niphates digitalis* | 8 | -17.4 | 0.4 | -18.1 | -16.9 | 10 | -17.6 | 1.2 | -19.6 | -16.4 |
|  |  | *Xestospongia muta* | 10 | -22.8 | 1.8 | -25.2 | -20.4 | 10 | -19.7 | 0.2 | -20.0 | -19.4 |
|  |  | total | 28 | -22.4 | 3.6 | -27.2 | -16.9 | 30 | -18.3 | 1.4 | -20.0 | -16.1 |
|  | cryptic sponge tissue | *Chondrilla sp.* | 2 | -26.3 | 0.9 | -26.9 | -25.7 | 3 | -17.2 | 0.5 | -17.5 | -16.6 |
|  |  | *Halisarca caerulea* | 3 | -17.9 | 1.4 | -19.6 | -16.9 | 4 | -12.6 | 2.8 | -16.8 | -10.7 |
|  |  | *Scopalina ruetzleri* | 3 | -22.1 | 0.6 | -22.8 | -21.5 | 5 | -21.4 | 0.7 | -22.3 | -20.8 |
|  |  | total | 8 | -21.6 | 3.6 | -26.9 | -16.9 | 12 | -17.4 | 4.2 | -22.3 | -10.7 |
|  | **sediment trap** | total | 5 | -18.7 | 0.3 | -19.1 | -18.3 | 14 | -20.0 | 1.9 | -26.1 | -18.1 |
