# Supplementary material for: Looking for the sponge loop: analyses of detritus on a Caribbean forereef using stable isotope and eDNA metabarcoding techniques
Source: PeerJ. 2024 Feb 23;12:e16970. doi: 10.7717/peerj.16970 (PMC10896084; doi:10.7717/peerj.16970)
Supplement: Table S4 — Composite samples are indicated in bold. [file peerj-12-16970-s008.docx]

|  |  |  | July 2018 | | | | | March 2019 | | | | |
| --- | --- | --- | --- | --- | --- | --- | --- | --- | --- | --- | --- | --- |
| category | sample type | species | n | ave | sd | min | max | n | ave | sd | min | max |
| detritus | **EAM** | total | 5 | 1.5 | 0.1 | 1.4 | 1.7 | 11 | 1.8 | 0.8 | 0.2 | 2.7 |
|  | **tray** | total | 0 | - | - | - | - | 3 | 1.9 | 0.5 | 1.6 | 2.5 |
| source | algae tissue | *Dictyota sp.* | 5 | 0.8 | 0.2 | 0.6 | 1.0 | 5 | 0.9 | 0.1 | 0.8 | 1.0 |
|  |  | *Halimeda sp.* | 0 | - | - | - | - | 3 | 1.7 | 0.2 | 1.5 | 1.9 |
|  |  | *Lobophora variegata* | 5 | 0.3 | 0.1 | 0.1 | 0.3 | 6 | 1.1 | 0.2 | 0.9 | 1.5 |
|  |  | total | 10 | 0.6 | 0.3 | 0.1 | 1.0 | 14 | 1.2 | 0.3 | 0.8 | 1.9 |
|  | BCM tissue | total | 3 | 1.0 | 0.3 | 0.7 | 1.2 | 5 | 1.0 | 0.4 | 0.6 | 1.6 |
|  | **herbivore feces** | *Acanthurus bahianus* | 3 | 1.2 | 0.2 | 1.0 | 1.3 | 4 | 1.1 | 0.5 | 0.6 | 1.8 |
|  |  | *Acanthurus coeruleus* | 2 | 0.9 | 0.2 | 0.8 | 1.0 | 1 | 0.6 | - | - | - |
|  |  | total | 5 | 1.1 | 0.2 | 0.8 | 1.3 | 5 | 1.0 | 0.5 | 0.6 | 1.8 |
|  | **spongivore feces** | *Holacanthus ciliaris* | 1 | 4.4 | - | - | - | 1 | 2.6 | - | - | - |
|  |  | *Pomacanthus paru* | 0 | - | - | - | - | 2 | 3.4 | 0.4 | 3.1 | 3.7 |
|  |  | total | 1 | 4.4 | - | - | - | 3 | 3.1 | 0.5 | 2.6 | 3.7 |
|  | emergent sponge tissue | *Aplysina cauliformis* | 10 | 3.0 | 0.7 | 2.0 | 4.3 | 10 | 0.7 | 1.3 | -2.1 | 2.1 |
|  |  | *Niphates digitalis* | 8 | 3.6 | 0.2 | 3.3 | 3.8 | 10 | 2.9 | 0.4 | 2.4 | 3.5 |
|  |  | *Xestospongia muta* | 10 | 3.8 | 0.4 | 3.3 | 4.4 | 10 | 3.5 | 0.5 | 3.1 | 4.4 |
|  |  | total | 28 | 3.4 | 0.6 | 2.0 | 4.4 | 30 | 2.4 | 1.5 | -2.1 | 4.4 |
|  | cryptic sponge tissue | *Chondrilla sp.* | 2 | 2.2 | 0.0 | 2.1 | 2.2 | 3 | 0.4 | 1.0 | -0.8 | 1.1 |
|  |  | *Halisarca caerulea* | 3 | 3.3 | 0.1 | 3.2 | 3.5 | 4 | 2.9 | 0.1 | 2.8 | 3.0 |
|  |  | *Scopalina ruetzleri* | 3 | 5.1 | 0.4 | 4.8 | 5.5 | 5 | 3.9 | 0.3 | 3.5 | 4.2 |
|  |  | total | 8 | 3.7 | 1.3 | 2.1 | 5.5 | 12 | 2.7 | 1.5 | -0.8 | 4.2 |
|  | **sediment trap** | total | 5 | -1.1 | 1.3 | -2.6 | 0.1 | 14 | 1.7 | 0.5 | 0.9 | 2.7 |
