# Supplementary material for: Looking for the sponge loop: analyses of detritus on a Caribbean forereef using stable isotope and eDNA metabarcoding techniques
Source: PeerJ. 2024 Feb 23;12:e16970. doi: 10.7717/peerj.16970 (PMC10896084; doi:10.7717/peerj.16970)
Supplement: Table S5 — For each taxonomic level, the threshold similarity score used for assignment is listed. Empty cells indicate that the taxonomy for that sample at that level did not meet the threshold or was ambiguous (i.e., matched two groups equally). [file peerj-12-16970-s009.docx]

| Sample | Expected Taxonomy | Detected Taxonomy | | | | | |
| --- | --- | --- | --- | --- | --- | --- | --- |
|  |  | Phylum (85%) | Class (90%) | Order (90%) | Family (95%) | Genus (98%) | Species (99%) |
| DI 1 | *Dictyota sp.* | (unassigned) | Phaeophyceae | Dictyotales | Dictyotaceae | *Dictyopteris* |  |
| DI 2 | *Dictyota sp.* | Rhodophyta | Florideophyceae | Ceramiales | Rhodomelaceae |  |  |
| DI 3 | *Dictyota sp.* | Arthropoda | Hexanauplia | Harpacticoida | Dactylopusiidae |  |  |
| DI 4 | *Dictyota sp.* | Rhodophyta | Florideophyceae | Ceramiales | Rhodomelaceae | *Lomentaria* |  |
| DI 5 | *Dictyota sp.* | Rhodophyta | Florideophyceae | Ceramiales | Rhodomelaceae | *Neosiphonia* |  |
| LV 1 | *Lobophora variegata* | (unassigned) | Phaeophyceae | Dictyotales | Dictyotaceae | *Lobophora* |  |
| LV 2 | *Lobophora variegata* | (unassigned) | Phaeophyceae | Dictyotales | Dictyotaceae | *Lobophora* |  |
| LV 3 | *Lobophora variegata* | (unassigned) | Phaeophyceae | Dictyotales | Dictyotaceae | *Lobophora* |  |
| LV 4 | *Lobophora variegata* | (unassigned) | Phaeophyceae | Dictyotales | Dictyotaceae | *Lobophora* |  |
| LV 5 | *Lobophora variegata* | (unassigned) | Phaeophyceae | Dictyotales | Dictyotaceae | *Lobophora* |  |
| CY 1 | red filamentous sp. unknown | Cyanobacteria | (unassigned) | Oscillatoriales | Oscillatoriaceae | *Moorea* | *producens* |
| CY 2 | red filamentous sp. unknown | Cyanobacteria | (unassigned) | Oscillatoriales | Oscillatoriaceae |  |  |
| CY 3 | red filamentous sp. unknown | Cyanobacteria | (unassigned) | Oscillatoriales | Oscillatoriaceae | *Moorea* | *producens* |
| CN 1 | *Chondrilla sp.* | Porifera | Demospongiae | Chondrillida | Chondrillidae | *Chondrilla* |  |
| CN 2 | *Chondrilla sp.* | Porifera | Demospongiae | Chondrillida | Chondrillidae | *Chondrilla* |  |
| CN 3 | *Chondrilla sp.* | Porifera | Demospongiae | Chondrillida | Chondrillidae | *Chondrilla* |  |
| HC 1 | *Halisarca caerulea* | Porifera | Demospongiae | Dendroceratida | Halisarcidae* |  |  |
| HC 2 | *Halisarca caerulea* | Porifera | Demospongiae | Dendroceratida | Halisarcidae |  |  |
| HC 3 | *Halisarca caerulea* | Porifera | Demospongiae | Dendroceratida | Halisarcidae |  |  |
| SR 1 | *Scopalina ruetzleri* | Porifera | Demospongiae | Bubarida | Dictyonellidae | *Scopalina* | *ruetzleri* |
| SR 2 | *Scopalina ruetzleri* | Porifera | Demospongiae | Bubarida | Dictyonellidae | *Scopalina* | *ruetzleri* |
| SR 3 | *Scopalina ruetzleri* | Porifera | Demospongiae | Bubarida | Dictyonellidae | *Scopalina* | *ruetzleri* |
| XM 1 | *Xestospongia muta* | Porifera | Demospongiae | Haplosclerida | Petrosiidae | *Xestospongia* |  |
| XM 2 | *Xestospongia muta* | Porifera | Demospongiae | Haplosclerida | Petrosiidae | *Xestospongia* |  |
| XM 3 | *Xestospongia muta* | Porifera | Demospongiae | Haplosclerida | Petrosiidae | *Xestospongia* | *muta* |
| XM 4 | *Xestospongia muta* | Porifera | Demospongiae | Haplosclerida | Petrosiidae | *Xestospongia* |  |
| XM 5 | *Xestospongia muta* | Porifera | Demospongiae | Haplosclerida | Petrosiidae | *Xestospongia* | *muta* |
|  | *the sample matched to the expected genus (*Halisarca*) just below our genus-level threshold with a score of 0.96 | | | | | | |
