# Supplementary material for: Looking for the sponge loop: analyses of detritus on a Caribbean forereef using stable isotope and eDNA metabarcoding techniques
Source: PeerJ. 2024 Feb 23;12:e16970. doi: 10.7717/peerj.16970 (PMC10896084; doi:10.7717/peerj.16970)
Supplement: Table S6 — Classification includes phylum (P), class (C), order (O), family (F), genus (G), and species (S), where applicable. EAM = detritus suctioned from epilithic algal matrix in July 2018 (J18; n = 5) and March 2019 (M19, n = 6). sed. trap = particulates captured in sediment traps in July 2018 (n = 5) and March 2019 ( n = 7). tray = detritus sampled from trays placed under reef overhangs in March 2019 (n = 2). h’vore fec = feces collected from herbivorous fishes in 2018 (n = 5) and 2019 (n = 7). s’vore fec = feces collected from spongivore fishes in 2018 ( n = 1) and 2019 (n = 4). [file peerj-12-16970-s010.docx]

| Classification | EAM | | sed. trap | | tray | h'vore fec | | s'vore fec | |
| --- | --- | --- | --- | --- | --- | --- | --- | --- | --- |
|  | J18 | M19 | J18 | M19 | M19 | J18 | M19 | J18 | M19 |
| P: Cyanobacteria; O: Chroococcales; F: Aphanothecaceae; S: *Crocosphaera subtropica* | 20% | - | - | - | 50% | - | - | - | - |
| P: Cyanobacteria; O: Chroococcales; F: Aphanothecaceae; S: *Crocosphaera watsonii* | 40% | - | - | - | - | - | 14% | - | - |
| P: Cyanobacteria; O: Chroococcales; F: Aphanothecaceae; G: *Crocosphaera* | 40% | - | - | - | 50% | - | 14% | - | - |
| P: Cyanobacteria; O: Chroococcales; F: Aphanothecaceae; G: *Rippkaea* | 40% | - | - | - | - | - | - | - | - |
| P: Cyanobacteria; O: Chroococcales; F: Aphanothecaceae; S: *Zehria floridana* | 20% | - | - | - | - | - | - | - | - |
| P: Cyanobacteria; O: Chroococcales; F: Aphanothecaceae; G: *Zehria* | 40% | - | - | - | - | - | - | - | - |
| P: Cyanobacteria; O: Chroococcales; F: Aphanothecaceae | 100% | 17% | 60% | 86% | 100% | - | 57% | - | - |
| P: Cyanobacteria; O: Chroococcales; F: Chroococcaceae | 40% | - | - | - | - | - | 14% | - | - |
| P: Cyanobacteria; O: Chroococcales; F: Cyanobacteriaceae | 40% | - | - | - | 50% | - | - | - | - |
| P: Cyanobacteria; O: Chroococcales; F: Gomphosphaeriaceae | 40% | - | - | - | 50% | - | - | - | - |
| P: Cyanobacteria; O: Chroococcales | 100% | 67% | 80% | 100% | 100% | 60% | 86% | - | - |
| P: Cyanobacteria; O: Chroococcidiopsidales | 20% | - | - | - | - | - | 14% | - | - |
| P: Cyanobacteria; O: Nostocales; F: Aphanizomenonaceae; G: *Nodularia* | 20% | - | - | - | - | - | - | - | - |
| P: Cyanobacteria; O: Nostocales; F: Aphanizomenonaceae | 40% | - | - | - | 100% | - | - | - | - |
| P: Cyanobacteria; O: Nostocales; F: Calotrichaceae; S: *Calothrix parasitica* | 40% | - | - | - | - | - | - | - | - |
| P: Cyanobacteria; O: Nostocales; F: Calotrichaceae; G: *Calothrix* | 40% | - | - | - | - | - | - | - | - |
| P: Cyanobacteria; O: Nostocales; F: Calotrichaceae | 40% | - | - | - | - | - | - | - | - |
| P: Cyanobacteria; O: Nostocales; F: Gloeotrichiaceae | 20% | - | - | - | - | - | - | - | - |
| P: Cyanobacteria; O: Nostocales; F: Nostocaceae; G: *Nostoc* | 20% | - | - | - | - | - | - | - | - |
| P: Cyanobacteria; O: Nostocales; F: Nostocaceae | 40% | - | - | - | 50% | - | - | - | - |
| P: Cyanobacteria; O: Nostocales; F: Rivulariaceae; G: *Microchaete* | 20% | - | - | - | - | - | - | - | - |
| P: Cyanobacteria; O: Nostocales; F: Rivulariaceae | 40% | - | - | 29% | 50% | - | - | - | - |
| P: Cyanobacteria; O: Nostocales; F: Scytonemataceae | 40% | - | - | - | 100% | - | 29% | - | - |
| P: Cyanobacteria; O: Nostocales; F: Stigonemataceae | 40% | - | - | - | - | - | - | - | - |
| P: Cyanobacteria; O: Nostocales; F: Symphyonemataceae | 20% | - | - | - | - | - | - | - | - |
| P: Cyanobacteria; O: Nostocales; F: Tolypothrichaceae | 40% | - | - | - | - | - | - | - | - |
| P: Cyanobacteria; O: Nostocales | 100% | 67% | 60% | 86% | 100% | 40% | 86% | - | - |
| P: Cyanobacteria; O: Oscillatoriales; F: Coleofasciculaceae | 40% | - | - | - | 50% | - | - | - | - |
| P: Cyanobacteria; O: Oscillatoriales; F: Gomontiellaceae; S: *Hormoscilla spongeliae* | 40% | - | - | - | - | - | - | - | - |
| P: Cyanobacteria; O: Oscillatoriales; F: Gomontiellaceae; G: *Hormoscilla* | 40% | - | - | - | - | - | - | - | - |
| P: Cyanobacteria; O: Oscillatoriales; F: Gomontiellaceae | 40% | - | - | - | - | - | - | - | - |
| P: Cyanobacteria; O: Oscillatoriales; F: Microcoleaceae; S: *Hydrocoleum lyngbyaceum* | 20% | - | - | - | - | - | - | - | - |
| P: Cyanobacteria; O: Oscillatoriales; F: Microcoleaceae; S: *Trichodesmium erythraeum* | 40% | - | - | - | - | - | - | - | - |
| P: Cyanobacteria; O: Oscillatoriales; F: Microcoleaceae; S: *Trichodesmium thiebautii* | 40% | - | - | - | 50% | - | 14% | - | - |
| P: Cyanobacteria; O: Oscillatoriales; F: Microcoleaceae; G: *Trichodesmium* | 40% | - | - | - | 50% | - | 14% | - | - |
| P: Cyanobacteria; O: Oscillatoriales; F: Microcoleaceae | 100% | 33% | 80% | 71% | 50% | 40% | 29% | - | - |
| P: Cyanobacteria; O: Oscillatoriales; F: Oscillatoriaceae; S: *Moorea producens* | 40% | - | - | - | 50% | - | - | - | - |
| P: Cyanobacteria; O: Oscillatoriales; F: Oscillatoriaceae; G: *Moorea* | 20% | - | - | - | 50% | - | - | - | - |
| P: Cyanobacteria; O: Oscillatoriales; F: Oscillatoriaceae; S: *Neolyngbya granulosa* | 20% | - | - | - | - | - | - | - | - |
| P: Cyanobacteria; O: Oscillatoriales; F: Oscillatoriaceae; G: *Neolyngbya* | 20% | - | - | - | - | - | - | - | - |
| P: Cyanobacteria; O: Oscillatoriales; F: Oscillatoriaceae; G: *Oscillatoria* | 20% | - | - | - | - | - | - | - | - |
| P: Cyanobacteria; O: Oscillatoriales; F: Oscillatoriaceae | 60% | 17% | 40% | 57% | 50% | - | 29% | - | - |
| P: Cyanobacteria; O: Oscillatoriales | 100% | 50% | 80% | 100% | 100% | 60% | 86% | - | - |
| P: Cyanobacteria; O: Pleurocapsales; F: Dermocarpellaceae | 40% | - | - | - | - | - | 14% | - | - |
| P: Cyanobacteria; O: Pleurocapsales; F: Hyellaceae; G: *Chroococcopsis* | 20% | - | - | - | - | - | - | - | - |
| P: Cyanobacteria; O: Pleurocapsales; F: Hyellaceae | 40% | - | - | - | 50% | - | 14% | - | - |
| P: Cyanobacteria; O: Pleurocapsales; F: Xenococcaceae; G: *Foliisarcina* | 40% | - | - | - | 50% | - | 29% | - | - |
| P: Cyanobacteria; O: Pleurocapsales; F: Xenococcaceae | 40% | - | - | - | 100% | - | 29% | - | - |
| P: Cyanobacteria; O: Pleurocapsales | 100% | 67% | 100% | 100% | 100% | 60% | 100% | - | - |
| P: Cyanobacteria; O: Spirulinales; F: Spirulinaceae; S: *Spirulina subsalsa* | 40% | - | - | - | - | - | - | - | - |
| P: Cyanobacteria; O: Spirulinales; F: Spirulinaceae; G: *Spirulina* | 20% | - | - | - | - | - | - | - | - |
| P: Cyanobacteria; O: Spirulinales; F: Spirulinaceae | 40% | - | - | - | - | - | - | - | - |
| P: Cyanobacteria; O: Spirulinales | 40% | - | - | - | 50% | - | - | - | - |
| P: Cyanobacteria; O: Synechococcales; F: Acaryochloridaceae; G: *Acaryochloris* | 20% | - | - | - | - | - | - | - | - |
| P: Cyanobacteria; O: Synechococcales; F: Acaryochloridaceae | 60% | - | - | 14% | 100% | - | 14% | - | - |
| P: Cyanobacteria; O: Synechococcales; F: Leptolyngbyaceae; S: *Leptolyngbya ectocarpi* | 40% | - | - | - | 50% | - | - | - | - |
| P: Cyanobacteria; O: Synechococcales; F: Leptolyngbyaceae; G: *Leptolyngbya* | 40% | - | - | - | 100% | - | - | - | - |
| P: Cyanobacteria; O: Synechococcales; F: Leptolyngbyaceae | 80% | 33% | 60% | 86% | 50% | - | 29% | - | - |
| P: Cyanobacteria; O: Synechococcales; F: Prochloraceae; S: *Prochlorococcus marinus* | 40% | - | - | - | 100% | - | 14% | - | - |
| P: Cyanobacteria; O: Synechococcales; F: Prochloraceae | 20% | - | 80% | - | 50% | 20% | - | - | - |
| P: Cyanobacteria; O: Synechococcales; F: Prochlorotrichaceae | 40% | - | - | - | 50% | - | - | - | - |
| P: Cyanobacteria; O: Synechococcales; F: Pseudanabaenaceae; S: *Limnothrix rosea* | 40% | - | - | - | 50% | - | 14% | - | - |
| P: Cyanobacteria; O: Synechococcales; F: Pseudanabaenaceae; G: *Limnothrix* | 40% | - | - | - | 50% | - | 14% | - | - |
| P: Cyanobacteria; O: Synechococcales; F: Pseudanabaenaceae | 40% | - | - | 14% | 50% | - | 29% | - | - |
| P: Cyanobacteria; O: Synechococcales; F: Romeriaceae | 40% | - | - | - | - | - | - | - | - |
| P: Cyanobacteria; O: Synechococcales; F: Synechococcaceae | 100% | 33% | 100% | 100% | - | 60% | 43% | 100% | - |
| P: Cyanobacteria; O: Synechococcales | 100% | 50% | 100% | 100% | 100% | 40% | 71% | 100% | - |
| P: Cyanobacteria | 100% | 83% | 100% | 100% | 100% | 80% | 100% | 100% | 50% |
| P: Bacillariophyta; C: Bacillariophyceae; O: Bacillariales; F: Bacillariaceae; S: *Bacillaria paxillifer* | 20% | - | - | - | 50% | - | - | - | - |
| P: Bacillariophyta; C: Bacillariophyceae; O: Bacillariales; F: Bacillariaceae; G: *Bacillaria* | 20% | 33% | 20% | 71% | - | - | - | - | - |
| P: Bacillariophyta; C: Bacillariophyceae; O: Bacillariales; F: Bacillariaceae; S: *Cylindrotheca closterium* | 60% | - | 40% | 29% | 50% | - | 14% | - | - |
| P: Bacillariophyta; C: Bacillariophyceae; O: Bacillariales; F: Bacillariaceae; G: *Cylindrotheca* | 60% | 17% | 20% | 14% | 50% | - | - | - | - |
| P: Bacillariophyta; C: Bacillariophyceae; O: Bacillariales; F: Bacillariaceae; S: *Fragilariopsis cylindrus* | 40% | - | - | - | 50% | - | 14% | - | - |
| P: Bacillariophyta; C: Bacillariophyceae; O: Bacillariales; F: Bacillariaceae; G: *Fragilariopsis* | 40% | - | - | - | 100% | - | 29% | - | - |
| P: Bacillariophyta; C: Bacillariophyceae; O: Bacillariales; F: Bacillariaceae; S: *Nitzschia palea* | 40% | - | - | - | 100% | - | 14% | - | - |
| P: Bacillariophyta; C: Bacillariophyceae; O: Bacillariales; F: Bacillariaceae; S: *Nitzschia pusilla* | 20% | - | - | - | - | - | - | - | - |
| P: Bacillariophyta; C: Bacillariophyceae; O: Bacillariales; F: Bacillariaceae; S: *Nitzschia thermalis* | 40% | - | - | - | 100% | - | 14% | - | - |
| P: Bacillariophyta; C: Bacillariophyceae; O: Bacillariales; F: Bacillariaceae; G: *Nitzschia* | 100% | 17% | 100% | 100% | 100% | - | 14% | - | - |
| P: Bacillariophyta; C: Bacillariophyceae; O: Bacillariales; F: Bacillariaceae; S: *Psammodictyon pustulatum* | - | 17% | - | - | - | - | - | - | - |
| P: Bacillariophyta; C: Bacillariophyceae; O: Bacillariales; F: Bacillariaceae; G: *Psammodictyon* | - | 17% | - | - | - | - | 14% | - | - |
| P: Bacillariophyta; C: Bacillariophyceae; O: Bacillariales; F: Bacillariaceae; S: *Pseudo-nitzschia seriata* | 40% | - | - | - | 50% | - | - | - | - |
| P: Bacillariophyta; C: Bacillariophyceae; O: Bacillariales; F: Bacillariaceae; G: *Pseudo-nitzschia* | 80% | - | 40% | 14% | 50% | - | 14% | 100% | - |
| P: Bacillariophyta; C: Bacillariophyceae; O: Bacillariales; F: Bacillariaceae; G: *Tryblionella* | 20% | 17% | - | 14% | - | - | - | - | - |
| P: Bacillariophyta; C: Bacillariophyceae; O: Bacillariales; F: Bacillariaceae | 100% | 50% | 100% | 100% | 100% | 20% | 71% | - | - |
| P: Bacillariophyta; C: Bacillariophyceae; O: Bacillariales | 100% | 100% | 100% | 100% | 100% | 100% | 86% | 100% | 25% |
| P: Bacillariophyta; C: Bacillariophyceae; O: Cocconeidales; F: Achnanthidiaceae; G: *Astartiella* | - | 17% | 20% | 43% | - | - | - | - | - |
| P: Bacillariophyta; C: Bacillariophyceae; O: Cocconeidales; F: Achnanthidiaceae | 20% | - | - | - | 50% | - | - | - | - |
| P: Bacillariophyta; C: Bacillariophyceae; O: Cocconeidales; F: Cocconeidaceae | 40% | 17% | 20% | 71% | - | - | 14% | - | - |
| P: Bacillariophyta; C: Bacillariophyceae; O: Cocconeidales | 80% | - | - | 71% | 50% | - | 29% | - | - |
| P: Bacillariophyta; C: Bacillariophyceae; O: Cymbellales; F: Cymbellaceae | 40% | - | - | - | - | - | - | - | - |
| P: Bacillariophyta; C: Bacillariophyceae; O: Cymbellales; F: Gomphonemataceae; S: *Didymosphenia geminata* | 20% | - | - | - | - | - | - | - | - |
| P: Bacillariophyta; C: Bacillariophyceae; O: Cymbellales; F: Gomphonemataceae; G: *Didymosphenia* | 40% | - | - | - | 100% | - | - | - | - |
| P: Bacillariophyta; C: Bacillariophyceae; O: Cymbellales; F: Gomphonemataceae; G: *Gomphoneis* | 40% | - | - | - | 50% | - | - | - | - |
| P: Bacillariophyta; C: Bacillariophyceae; O: Cymbellales; F: Gomphonemataceae | 40% | - | - | - | 50% | - | 29% | - | - |
| P: Bacillariophyta; C: Bacillariophyceae; O: Cymbellales | 60% | 17% | - | 43% | 50% | - | 43% | - | - |
| P: Bacillariophyta; C: Bacillariophyceae; O: Eunotiales; F: Eunotiaceae; S: *Eunotia naegelii* | 40% | - | - | - | 50% | - | 14% | - | - |
| P: Bacillariophyta; C: Bacillariophyceae; O: Eunotiales; F: Eunotiaceae; G: *Eunotia* | 40% | - | - | - | - | - | 14% | - | - |
| P: Bacillariophyta; C: Bacillariophyceae; O: Eunotiales; F: Eunotiaceae | 40% | 17% | - | - | 50% | - | 29% | - | - |
| P: Bacillariophyta; C: Bacillariophyceae; O: Eunotiales | 100% | 67% | 80% | 86% | 50% | 40% | 71% | - | - |
| P: Bacillariophyta; C: Bacillariophyceae; O: Mastogloiales; F: Achnanthaceae | 40% | - | - | - | - | - | - | - | - |
| P: Bacillariophyta; C: Bacillariophyceae; O: Mastogloiales; F: Mastogloiaceae; G: *Mastogloia* | 40% | - | 20% | 43% | - | - | 14% | - | - |
| P: Bacillariophyta; C: Bacillariophyceae; O: Mastogloiales; F: Mastogloiaceae | 60% | 50% | 60% | 86% | - | - | 14% | - | - |
| P: Bacillariophyta; C: Bacillariophyceae; O: Mastogloiales | 100% | 33% | 60% | 71% | 50% | - | 43% | - | - |
| P: Bacillariophyta; C: Bacillariophyceae; O: Naviculales; F: Amphipleuraceae;  S: *Amphiprora paludosa* | 40% | - | - | - | 50% | - | - | - | - |
| P: Bacillariophyta; C: Bacillariophyceae; O: Naviculales; F: Amphipleuraceae; G: *Amphiprora* | 40% | - | - | - | 50% | - | - | - | - |
| P: Bacillariophyta; C: Bacillariophyceae; O: Naviculales; F: Amphipleuraceae;  S: *Halamphora coffeaeformis* | 40% | - | - | - | 100% | - | 29% | - | - |
| P: Bacillariophyta; C: Bacillariophyceae; O: Naviculales; F: Amphipleuraceae;  S: *Halamphora tenucostata* | 20% | 17% | - | - | - | - | - | - | - |
| P: Bacillariophyta; C: Bacillariophyceae; O: Naviculales; F: Amphipleuraceae; G: *Halamphora* | 40% | 33% | 20% | 29% | 100% | - | 57% | - | - |
| P: Bacillariophyta; C: Bacillariophyceae; O: Naviculales; F: Amphipleuraceae | 100% | 67% | 80% | 100% | 100% | 20% | 86% | - | 25% |
| P: Bacillariophyta; C: Bacillariophyceae; O: Naviculales; F: Diploneidaceae; G: *Diploneis* | 40% | - | - | 29% | - | - | 29% | - | - |
| P: Bacillariophyta; C: Bacillariophyceae; O: Naviculales; F: Diploneidaceae | 20% | - | 40% | 57% | - | - | 29% | - | - |
| P: Bacillariophyta; C: Bacillariophyceae; O: Naviculales; F: Naviculaceae; G: *Caloneis* | - | 17% | 20% | 14% | - | - | - | - | - |
| P: Bacillariophyta; C: Bacillariophyceae; O: Naviculales; F: Naviculaceae; S: *Dickieia ulvacea* | 40% | - | - | - | - | - | - | - | - |
| P: Bacillariophyta; C: Bacillariophyceae; O: Naviculales; F: Naviculaceae; G: *Dickieia* | 40% | - | - | - | 100% | - | - | - | - |
| P: Bacillariophyta; C: Bacillariophyceae; O: Naviculales; F: Naviculaceae; G: *Fistulifera* | 40% | - | - | - | - | - | 29% | - | - |
| P: Bacillariophyta; C: Bacillariophyceae; O: Naviculales; F: Naviculaceae; S: *Haslea nusantara* | 40% | - | - | - | 100% | - | 29% | - | - |
| P: Bacillariophyta; C: Bacillariophyceae; O: Naviculales; F: Naviculaceae; G: *Haslea* | 60% | - | 80% | 14% | 50% | - | 29% | - | - |
| P: Bacillariophyta; C: Bacillariophyceae; O: Naviculales; F: Naviculaceaevicula arenaria | 20% | - | - | - | - | - | 14% | - | - |
| P: Bacillariophyta; C: Bacillariophyceae; O: Naviculales; F: Naviculaceaevicula phyllepta | 40% | - | - | - | 100% | - | 29% | - | - |
| P: Bacillariophyta; C: Bacillariophyceae; O: Naviculales; F: Naviculaceae; G: *Navicula* | 80% | 33% | 100% | 100% | 100% | 20% | 86% | - | - |
| P: Bacillariophyta; C: Bacillariophyceae; O: Naviculales; F: Naviculaceae; S: *Seminavis robusta* | 80% | 17% | 80% | 57% | - | 20% | 29% | - | - |
| P: Bacillariophyta; C: Bacillariophyceae; O: Naviculales; F: Naviculaceae; G: *Seminavis* | 40% | - | - | - | - | 20% | 29% | - | - |
| P: Bacillariophyta; C: Bacillariophyceae; O: Naviculales; F: Naviculaceae | 100% | 50% | 100% | 100% | 100% | 80% | 86% | - | - |
| P: Bacillariophyta; C: Bacillariophyceae; O: Naviculales; F: Phaeodactylaceae;  S: *Phaeodactylum tricornutum* | 40% | - | - | - | 100% | - | - | - | - |
| P: Bacillariophyta; C: Bacillariophyceae; O: Naviculales; F: Phaeodactylaceae; G: *Phaeodactylum* | 40% | - | - | - | 100% | - | - | - | - |
| P: Bacillariophyta; C: Bacillariophyceae; O: Naviculales; F: Phaeodactylaceae | 40% | 17% | 40% | 57% | 100% | - | 14% | - | - |
| P: Bacillariophyta; C: Bacillariophyceae; O: Naviculales; F: Plagiotropidaceae | 20% | - | - | - | - | - | - | - | - |
| P: Bacillariophyta; C: Bacillariophyceae; O: Naviculales; F: Pleurosigmataceae; G: *Pleurosigma* | - | 50% | 40% | 86% | - | - | - | - | - |
| P: Bacillariophyta; C: Bacillariophyceae; O: Naviculales; F: Pleurosigmataceae; G: *Rhoicosigma* | 20% | - | - | 14% | - | - | - | - | - |
| P: Bacillariophyta; C: Bacillariophyceae; O: Naviculales; F: Pleurosigmataceae | 60% | 33% | 20% | 14% | 100% | - | - | - | - |
| P: Bacillariophyta; C: Bacillariophyceae; O: Naviculales; F: Proschkiniaceae | 20% | - | 80% | 29% | - | - | - | - | - |
| P: Bacillariophyta; C: Bacillariophyceae; O: Naviculales; F: Scoliotropidaceae; G: *Biremis* | - | 17% | - | - | - | - | - | - | - |
| P: Bacillariophyta; C: Bacillariophyceae; O: Naviculales; F: Scoliotropidaceae | - | 17% | - | - | - | - | - | - | - |
| P: Bacillariophyta; C: Bacillariophyceae; O: Naviculales; F: Stauroneidaceae; G: *Stauroneis* | 20% | 17% | - | 29% | - | - | - | - | - |
| P: Bacillariophyta; C: Bacillariophyceae; O: Naviculales; F: Stauroneidaceae | 60% | 17% | - | 29% | - | - | 29% | - | - |
| P: Bacillariophyta; C: Bacillariophyceae; O: Naviculales | 100% | 100% | 100% | 100% | 100% | 80% | 100% | 100% | 25% |
| P: Bacillariophyta; C: Bacillariophyceae; O: Rhopalodiales; F: Rhopalodiaceae; G: *Rhopalodia* | 60% | 17% | 20% | 57% | - | - | - | - | - |
| P: Bacillariophyta; C: Bacillariophyceae; O: Rhopalodiales; F: Rhopalodiaceae | 40% | - | - | 14% | - | 20% | - | - | - |
| P: Bacillariophyta; C: Bacillariophyceae; O: Rhopalodiales | 60% | - | - | - | 100% | - | - | - | - |
| P: Bacillariophyta; C: Bacillariophyceae; O: Surirellales; F: Auriculaceae | 40% | - | - | 29% | - | - | - | - | - |
| P: Bacillariophyta; C: Bacillariophyceae; O: Surirellales; F: Surirellaceae; S: *Cymatopleura solea* | 40% | - | - | - | 100% | - | - | - | - |
| P: Bacillariophyta; C: Bacillariophyceae; O: Surirellales; F: Surirellaceae; G: *Cymatopleura* | 40% | - | - | - | 50% | - | 14% | - | - |
| P: Bacillariophyta; C: Bacillariophyceae; O: Surirellales; F: Surirellaceae; G: *Surirella* | 20% | 17% | 20% | 14% | - | 20% | - | - | - |
| P: Bacillariophyta; C: Bacillariophyceae; O: Surirellales; F: Surirellaceae | 60% | - | 60% | - | 50% | - | 14% | - | - |
| P: Bacillariophyta; C: Bacillariophyceae; O: Surirellales | 40% | - | - | 14% | 50% | - | 14% | - | - |
| P: Bacillariophyta; C: Bacillariophyceae; O: Thalassiophysales; F: Catenulaceae; S: *Amphora obtusa* | 20% | - | - | - | - | - | - | - | - |
| P: Bacillariophyta; C: Bacillariophyceae; O: Thalassiophysales; F: Catenulaceae; G: *Amphora* | 40% | 17% | 40% | 57% | - | - | 14% | - | - |
| P: Bacillariophyta; C: Bacillariophyceae; O: Thalassiophysales; F: Catenulaceae | 80% | 17% | 80% | 43% | 50% | - | 14% | - | - |
| P: Bacillariophyta; C: Bacillariophyceae; O: Thalassiophysales; F: Thalassiophysaceae; S: *Thalassiophysa hyalina* | 20% | - | 20% | - | - | - | - | - | - |
| P: Bacillariophyta; C: Bacillariophyceae; O: Thalassiophysales | 100% | 67% | 60% | 100% | 100% | 20% | 14% | - | - |
| P: Bacillariophyta; C: Bacillariophyceae; F: Entomoneidaceae; G: *Entomoneis* | 60% | 17% | 100% | 86% | 100% | 20% | 29% | - | - |
| P: Bacillariophyta; C: Bacillariophyceae; F: Entomoneidaceae | 100% | 67% | 100% | 100% | 100% | 40% | 86% | 100% | - |
| P: Bacillariophyta; C: Bacillariophyceae | 100% | 83% | 100% | 100% | 100% | 80% | 86% | 100% | - |
| P: Bacillariophyta; C: Coscinodiscophyceae; O: Chaetocerotales; F: Attheyaceae | 40% | - | - | - | 50% | - | 14% | - | - |
| P: Bacillariophyta; C: Coscinodiscophyceae; O: Chaetocerotales; F: Chaetocerotaceae; S: *Chaetoceros calcitrans* | 40% | - | - | - | 50% | - | - | - | - |
| P: Bacillariophyta; C: Coscinodiscophyceae; O: Chaetocerotales; F: Chaetocerotaceae; S: *Chaetoceros muellerii* | 20% | - | - | - | 50% | - | - | - | - |
| P: Bacillariophyta; C: Coscinodiscophyceae; O: Chaetocerotales; F: Chaetocerotaceae; S: *Chaetoceros simplex* | 20% | - | - | - | 50% | - | - | - | - |
| P: Bacillariophyta; C: Coscinodiscophyceae; O: Chaetocerotales; F: Chaetocerotaceae; S: *Chaetoceros socialis* | 20% | - | - | - | - | - | - | - | - |
| P: Bacillariophyta; C: Coscinodiscophyceae; O: Chaetocerotales; F: Chaetocerotaceae; G: *Chaetoceros* | 40% | - | - | - | 100% | - | 29% | - | - |
| P: Bacillariophyta; C: Coscinodiscophyceae; O: Chaetocerotales; F: Chaetocerotaceae | 60% | - | 60% | 57% | 50% | - | 29% | - | - |
| P: Bacillariophyta; C: Coscinodiscophyceae; O: Chaetocerotales | 100% | 50% | 100% | 100% | 100% | - | 29% | - | - |
| P: Bacillariophyta; C: Coscinodiscophyceae; O: Coscinodiscales; F: Coscinodiscaceae; S: *Coscinodiscus radiatus* | 40% | - | - | - | - | - | - | - | - |
| P: Bacillariophyta; C: Coscinodiscophyceae; O: Coscinodiscales; F: Coscinodiscaceae; G: *Coscinodiscus* | 40% | - | - | - | - | - | - | - | - |
| P: Bacillariophyta; C: Coscinodiscophyceae; O: Coscinodiscales; F: Coscinodiscaceae | 40% | - | - | - | 100% | - | 29% | - | - |
| P: Bacillariophyta; C: Coscinodiscophyceae; O: Coscinodiscales; F: Hemidiscaceae; S: *Actinocyclus subtilis* | 40% | - | - | - | 100% | - | - | - | - |
| P: Bacillariophyta; C: Coscinodiscophyceae; O: Coscinodiscales; F: Hemidiscaceae; G: *Actinocyclus* | 100% | 17% | 80% | 71% | 100% | 20% | 14% | - | - |
| P: Bacillariophyta; C: Coscinodiscophyceae; O: Coscinodiscales; F: Hemidiscaceae | 100% | - | 80% | 100% | 100% | 20% | 14% | 100% | - |
| P: Bacillariophyta; C: Coscinodiscophyceae; O: Coscinodiscales | 80% | 67% | 40% | 100% | 100% | 40% | 71% | - | - |
| P: Bacillariophyta; C: Coscinodiscophyceae; O: Leptocylindrales; F: Leptocylindraceae | 20% | - | - | - | - | - | - | - | - |
| P: Bacillariophyta; C: Coscinodiscophyceae; O: Leptocylindrales | 40% | - | - | - | - | - | - | - | - |
| P: Bacillariophyta; C: Coscinodiscophyceae; O: Melosirales; F: Hyalodiscaceae; S: *Podosira stelligera* | 40% | - | - | - | - | - | - | - | - |
| P: Bacillariophyta; C: Coscinodiscophyceae; O: Melosirales; F: Hyalodiscaceae; G: *Podosira* | 40% | - | - | - | 50% | - | - | - | - |
| P: Bacillariophyta; C: Coscinodiscophyceae; O: Melosirales; F: Hyalodiscaceae | 40% | - | - | - | - | - | - | - | - |
| P: Bacillariophyta; C: Coscinodiscophyceae; O: Melosirales; F: Stephanopyxidaceae | 20% | - | 20% | - | 50% | - | - | - | - |
| P: Bacillariophyta; C: Coscinodiscophyceae; O: Melosirales | 40% | - | - | - | - | - | 14% | - | - |
| P: Bacillariophyta; C: Coscinodiscophyceae; O: Paraliales; F: Paraliaceae; S: *Paralia longispina* | 60% | 33% | 20% | 71% | - | 40% | - | - | - |
| P: Bacillariophyta; C: Coscinodiscophyceae; O: Paraliales; F: Paraliaceae; S: *Paralia sulcata* | 40% | - | - | - | 100% | - | - | - | - |
| P: Bacillariophyta; C: Coscinodiscophyceae; O: Paraliales; F: Paraliaceae; G: *Paralia* | 40% | - | - | - | 50% | - | - | - | - |
| P: Bacillariophyta; C: Coscinodiscophyceae; O: Paraliales; F: Paraliaceae | 40% | - | - | - | 100% | - | - | - | - |
| P: Bacillariophyta; C: Coscinodiscophyceae; O: Paraliales | 40% | - | - | - | 100% | - | - | - | - |
| P: Bacillariophyta; C: Coscinodiscophyceae; O: Rhizosoleniales; F: Rhizosoleniaceae; G: *Rhizosolenia* | 20% | - | - | - | - | - | - | - | - |
| P: Bacillariophyta; C: Coscinodiscophyceae; O: Rhizosoleniales; F: Rhizosoleniaceae | 40% | - | 40% | - | 50% | - | - | - | - |
| P: Bacillariophyta; C: Coscinodiscophyceae; O: Rhizosoleniales | 40% | - | 20% | - | - | - | 14% | - | - |
| P: Bacillariophyta; C: Coscinodiscophyceae; O: Thalassiosirales; F: Stephanodiscaceae; G: *Cyclotella* | 20% | - | 20% | - | - | - | - | - | - |
| P: Bacillariophyta; C: Coscinodiscophyceae; O: Thalassiosirales; F: Thalassiosiraceae; S: *Conticribra weissflogii* | 40% | - | - | - | 50% | - | - | - | - |
| P: Bacillariophyta; C: Coscinodiscophyceae; O: Thalassiosirales; F: Thalassiosiraceae; G: *Conticribra* | 40% | - | - | - | 100% | - | - | - | - |
| P: Bacillariophyta; C: Coscinodiscophyceae; O: Thalassiosirales; F: Thalassiosiraceae; S: *Thalassiosira pseudonana* | 20% | - | - | - | 50% | - | - | - | - |
| P: Bacillariophyta; C: Coscinodiscophyceae; O: Thalassiosirales; F: Thalassiosiraceae; G: *Thalassiosira* | 40% | - | 40% | - | 50% | - | - | - | - |
| P: Bacillariophyta; C: Coscinodiscophyceae; O: Thalassiosirales; F: Thalassiosiraceae | 40% | - | - | - | 100% | - | 29% | - | - |
| P: Bacillariophyta; C: Coscinodiscophyceae; O: Thalassiosirales | 60% | 17% | 80% | 29% | 50% | 20% | 43% | - | 25% |
| P: Bacillariophyta; C: Coscinodiscophyceae | 60% | 50% | 20% | 86% | 100% | 20% | 71% | - | - |
| P: Bacillariophyta; C: Fragilariophyceae; O: Cyclophorales; F: Cyclophoraceae | - | 17% | 20% | 14% | 50% | - | - | - | - |
| P: Bacillariophyta; C: Fragilariophyceae; O: Cyclophorales | 40% | - | - | - | 50% | - | - | - | - |
| P: Bacillariophyta; C: Fragilariophyceae; O: Fragilariales; F: Fragilariaceae; G: *Asterionella* | 40% | - | - | - | 100% | - | - | - | - |
| P: Bacillariophyta; C: Fragilariophyceae; O: Fragilariales; F: Fragilariaceae; S: *Asterionellopsis glacialis* | 40% | - | - | - | 50% | - | 14% | - | - |
| P: Bacillariophyta; C: Fragilariophyceae; O: Fragilariales; F: Fragilariaceae; G: *Asterionellopsis* | 40% | - | - | - | 100% | - | 29% | - | - |
| P: Bacillariophyta; C: Fragilariophyceae; O: Fragilariales; F: Fragilariaceae; S: *Bleakeleya notata* | 20% | 17% | 20% | 57% | - | - | 14% | - | - |
| P: Bacillariophyta; C: Fragilariophyceae; O: Fragilariales; F: Fragilariaceae; G: *Bleakeleya* | 20% | 17% | 100% | 71% | - | - | - | - | - |
| P: Bacillariophyta; C: Fragilariophyceae; O: Fragilariales; F: Fragilariaceae; S: *Diatoma tenuis* | 40% | - | - | - | - | - | 14% | - | - |
| P: Bacillariophyta; C: Fragilariophyceae; O: Fragilariales; F: Fragilariaceae; G: *Diatoma* | 20% | - | - | - | - | - | - | - | - |
| P: Bacillariophyta; C: Fragilariophyceae; O: Fragilariales; F: Fragilariaceae; S: *Gedaniella boltonii* | 20% | - | 20% | 14% | - | - | 29% | - | - |
| P: Bacillariophyta; C: Fragilariophyceae; O: Fragilariales; F: Fragilariaceae; S: *Neofragilaria montgomeryii* | 40% | 17% | - | 14% | - | - | - | - | - |
| P: Bacillariophyta; C: Fragilariophyceae; O: Fragilariales; F: Fragilariaceae; G: *Neofragilaria* | 40% | 33% | 20% | 43% | - | - | - | - | - |
| P: Bacillariophyta; C: Fragilariophyceae; O: Fragilariales; F: Fragilariaceae; G: *Perideraion* | - | 17% | - | - | - | - | - | - | - |
| P: Bacillariophyta; C: Fragilariophyceae; O: Fragilariales; F: Fragilariaceae; G: *Podocystis* | - | 17% | 20% | - | - | - | - | - | - |
| P: Bacillariophyta; C: Fragilariophyceae; O: Fragilariales; F: Fragilariaceae; S: *Synedra fragilaroides* | 40% | - | - | - | 100% | - | 29% | - | - |
| P: Bacillariophyta; C: Fragilariophyceae; O: Fragilariales; F: Fragilariaceae; S: *Synedra fulgens* | 40% | - | 80% | 14% | - | - | - | - | - |
| P: Bacillariophyta; C: Fragilariophyceae; O: Fragilariales; F: Fragilariaceae; G: *Synedra* | 100% | - | 40% | 71% | 100% | 20% | 57% | - | - |
| P: Bacillariophyta; C: Fragilariophyceae; O: Fragilariales; F: Fragilariaceae | 100% | 17% | 60% | 100% | 100% | 60% | 71% | - | - |
| P: Bacillariophyta; C: Fragilariophyceae; O: Fragilariales; F: Staurosiraceaenofrustulum shiloi | 40% | - | - | - | 50% | - | 14% | - | - |
| P: Bacillariophyta; C: Fragilariophyceae; O: Fragilariales; F: Staurosiraceae; G: *Nanofrustulum* | 40% | - | - | - | 50% | - | 14% | - | - |
| P: Bacillariophyta; C: Fragilariophyceae; O: Fragilariales; F: Staurosiraceae | 100% | 17% | 20% | 29% | 50% | 20% | 14% | - | - |
| P: Bacillariophyta; C: Fragilariophyceae; O: Fragilariales | 100% | 67% | 100% | 100% | 100% | 60% | 86% | - | - |
| P: Bacillariophyta; C: Fragilariophyceae; O: Licmophorales; F: Licmophoraceae; S: *Licmophora gracilis* | - | 17% | 20% | - | - | - | - | - | - |
| P: Bacillariophyta; C: Fragilariophyceae; O: Licmophorales; F: Licmophoraceae; G: *Licmophora* | 80% | 33% | 40% | 14% | 100% | - | 14% | - | - |
| P: Bacillariophyta; C: Fragilariophyceae; O: Licmophorales; F: Licmophoraceae; S: *Licmosphenia peragallioides* | 40% | 17% | 60% | - | - | - | - | - | - |
| P: Bacillariophyta; C: Fragilariophyceae; O: Licmophorales; F: Licmophoraceae | 100% | 33% | 80% | 100% | 100% | - | 29% | - | - |
| P: Bacillariophyta; C: Fragilariophyceae; O: Licmophorales; F: Ulnariaceae; S: *Hyalosynedra prasadii* | 20% | - | 40% | - | - | - | 14% | - | - |
| P: Bacillariophyta; C: Fragilariophyceae; O: Licmophorales; F: Ulnariaceae; G: *Hyalosynedra* | 60% | 17% | 60% | 71% | - | - | 43% | - | - |
| P: Bacillariophyta; C: Fragilariophyceae; O: Licmophorales; F: Ulnariaceae; S: *Ulnaria acus* | 40% | - | - | - | - | - | - | - | - |
| P: Bacillariophyta; C: Fragilariophyceae; O: Licmophorales; F: Ulnariaceae; G: *Ulnaria* | 20% | - | - | - | 50% | - | - | - | - |
| P: Bacillariophyta; C: Fragilariophyceae; O: Licmophorales; F: Ulnariaceae | 100% | - | 80% | 86% | 50% | - | 14% | - | - |
| P: Bacillariophyta; C: Fragilariophyceae; O: Licmophorales | 100% | 33% | 100% | 100% | 100% | - | 57% | - | 25% |
| P: Bacillariophyta; C: Fragilariophyceae; O: Rhabdonematales; F: Grammatophoraceae; S: *Grammatophora undulata* | - | 17% | - | 14% | - | - | - | - | - |
| P: Bacillariophyta; C: Fragilariophyceae; O: Rhabdonematales; F: Grammatophoraceae | 40% | - | 60% | 71% | 50% | - | 14% | - | - |
| P: Bacillariophyta; C: Fragilariophyceae; O: Rhabdonematales | 40% | - | 20% | 29% | 50% | - | - | - | - |
| P: Bacillariophyta; C: Fragilariophyceae; O: Striatellales; F: Striatellaceae; S: *Striatella unipunctata* | 20% | 17% | - | - | - | - | - | - | - |
| P: Bacillariophyta; C: Fragilariophyceae; O: Striatellales; F: Striatellaceae | 20% | - | 40% | 29% | - | - | - | - | - |
| P: Bacillariophyta; C: Fragilariophyceae; O: Striatellales | 100% | 17% | - | - | - | - | - | - | - |
| P: Bacillariophyta; C: Fragilariophyceae; O: Thalassionemales; F: Thalassionemataceae | 20% | - | - | 14% | 50% | - | - | - | - |
| P: Bacillariophyta; C: Fragilariophyceae; O: Thalassionemales | 20% | - | 20% | - | 50% | - | - | - | - |
| P: Bacillariophyta; C: Fragilariophyceae | 100% | 50% | 100% | 100% | 100% | 40% | 71% | - | 25% |
| P: Bacillariophyta; C: Mediophyceae; O: Anaulales; F: Anaulaceae; G: *Eunotogramma* | 40% | - | - | - | 100% | - | - | - | - |
| P: Bacillariophyta; C: Mediophyceae; O: Anaulales; F: Anaulaceae | 20% | - | - | - | 50% | - | 14% | - | - |
| P: Bacillariophyta; C: Mediophyceae; O: Anaulales | 100% | 33% | 60% | 71% | 50% | 40% | 43% | - | - |
| P: Bacillariophyta; C: Mediophyceae; O: Biddulphiales; F: Biddulphiaceae; S: *Biddulphia biddulphiana* | - | 17% | - | - | 50% | - | - | - | - |
| P: Bacillariophyta; C: Mediophyceae; O: Biddulphiales; F: Biddulphiaceae; S: *Biddulphia tridens* | - | 17% | - | - | 50% | - | - | - | - |
| P: Bacillariophyta; C: Mediophyceae; O: Biddulphiales; F: Biddulphiaceae; G: *Biddulphia* | 20% | - | - | - | 50% | - | - | - | - |
| P: Bacillariophyta; C: Mediophyceae; O: Biddulphiales; F: Biddulphiaceae; S: *Biddulphiopsis titiana* | 20% | - | 20% | 14% | - | - | 14% | - | - |
| P: Bacillariophyta; C: Mediophyceae; O: Biddulphiales; F: Biddulphiaceae | 40% | 33% | 20% | 71% | 100% | 20% | - | - | - |
| P: Bacillariophyta; C: Mediophyceae; O: Biddulphiales | 80% | 17% | 20% | 43% | 50% | - | 14% | - | - |
| P: Bacillariophyta; C: Mediophyceae; O: Cymatosirales; F: Cymatosiraceae; S: *Cymatosira lorenziana* | - | 17% | 20% | - | - | - | - | - | - |
| P: Bacillariophyta; C: Mediophyceae; O: Cymatosirales; F: Cymatosiraceae; S: *Plagiogrammopsis vanheurckii* | 40% | - | - | - | 100% | - | - | - | - |
| P: Bacillariophyta; C: Mediophyceae; O: Cymatosirales; F: Cymatosiraceae; G: *Plagiogrammopsis* | 40% | - | - | - | 50% | - | - | - | - |
| P: Bacillariophyta; C: Mediophyceae; O: Cymatosirales; F: Cymatosiraceae | 40% | - | - | - | 50% | - | 14% | - | - |
| P: Bacillariophyta; C: Mediophyceae; O: Cymatosirales | 100% | 17% | 60% | 29% | 50% | 20% | 29% | - | - |
| P: Bacillariophyta; C: Mediophyceae; O: Lithodesmiales | 40% | - | - | - | 50% | - | 14% | - | - |
| P: Bacillariophyta; C: Mediophyceae; O: Toxariales; F: Toxariaceae; S: *Toxarium undulatum* | 40% | - | - | - | 50% | - | - | - | - |
| P: Bacillariophyta; C: Mediophyceae; O: Toxariales; F: Toxariaceae; G: *Toxarium* | 60% | 17% | - | - | 100% | - | - | - | - |
| P: Bacillariophyta; C: Mediophyceae; O: Toxariales; F: Toxariaceae | 60% | - | - | - | 100% | - | - | - | - |
| P: Bacillariophyta; C: Mediophyceae; O: Toxariales | 100% | 67% | 80% | 100% | 50% | 40% | 14% | - | - |
| P: Bacillariophyta; C: Mediophyceae; O: Triceratiales; F: Plagiogrammaceae; G: *Dimeregramma* | 40% | 17% | 20% | - | - | - | - | - | - |
| P: Bacillariophyta; C: Mediophyceae; O: Triceratiales; F: Plagiogrammaceae; G: *Psammoneis* | 60% | 17% | 20% | - | - | - | - | - | - |
| P: Bacillariophyta; C: Mediophyceae; O: Triceratiales; F: Plagiogrammaceae | 60% | 17% | 20% | 57% | 100% | - | 57% | - | - |
| P: Bacillariophyta; C: Mediophyceae; O: Triceratiales; F: Triceratiaceae; S: *Lampriscus shadboltianum* | - | 17% | 20% | 57% | - | - | - | - | - |
| P: Bacillariophyta; C: Mediophyceae; O: Triceratiales; F: Triceratiaceae; G: *Lampriscus* | 20% | - | 20% | 57% | - | - | 14% | - | - |
| P: Bacillariophyta; C: Mediophyceae; O: Triceratiales; F: Triceratiaceae; S: *Triceratium dubium* | 40% | - | - | - | 50% | - | - | - | - |
| P: Bacillariophyta; C: Mediophyceae; O: Triceratiales; F: Triceratiaceae; S: *Triceratium gibbosum* | 40% | 33% | 20% | 71% | - | - | - | - | - |
| P: Bacillariophyta; C: Mediophyceae; O: Triceratiales; F: Triceratiaceae; S: *Triceratium pentacrinus* | 40% | 67% | - | 43% | - | - | - | - | - |
| P: Bacillariophyta; C: Mediophyceae; O: Triceratiales; F: Triceratiaceae; G: *Triceratium* | 60% | 33% | 20% | 57% | 50% | - | - | - | - |
| P: Bacillariophyta; C: Mediophyceae; O: Triceratiales; F: Triceratiaceae | 100% | 67% | 40% | 86% | 100% | - | 14% | - | - |
| P: Bacillariophyta; C: Mediophyceae; O: Triceratiales | 100% | 67% | 100% | 86% | 100% | 60% | 86% | - | - |
| P: Bacillariophyta; C: Mediophyceae | 80% | 17% | 40% | 43% | 50% | - | - | - | - |
| P: Bacillariophyta; S: *Prestauroneis integra* | - | 17% | - | - | - | - | - | - | - |
| P: Bacillariophyta | 100% | 100% | 100% | 100% | 100% | 80% | 86% | - | 50% |
| P: Chlorophyta; C: Chlorodendrophyceae; O: Chlorodendrales; F: Chlorodendraceae | - | 17% | - | - | - | - | - | - | - |
| P: Chlorophyta; C: Chlorophyceae; O: Chaetopeltidales | 20% | - | - | - | - | - | - | - | - |
| P: Chlorophyta; C: Chloropicophyceae; O: Chloropicales; F: Chloropicaceae; G: *Chloroparvula* | 20% | 17% | 80% | 43% | - | - | 14% | - | - |
| P: Chlorophyta; C: Chloropicophyceae; O: Chloropicales; F: Chloropicaceae; G: *Chloropicon* | 40% | 17% | 80% | 43% | 50% | 20% | - | - | - |
| P: Chlorophyta; C: Chloropicophyceae; O: Chloropicales; F: Chloropicaceae | 20% | - | 40% | 14% | 50% | - | 14% | - | - |
| P: Chlorophyta; C: Chloropicophyceae; O: Chloropicales | 40% | - | - | - | 100% | - | 29% | - | - |
| P: Chlorophyta; C: Mamiellophyceae; O: Mamiellales; F: Bathycoccaceae; S: *Bathycoccus prasinos* | 20% | 17% | - | 43% | 50% | - | 14% | - | - |
| P: Chlorophyta; C: Mamiellophyceae; O: Mamiellales; F: Mamiellaceae; S: *Micromonas commoda* | 20% | - | 20% | - | - | - | - | - | - |
| P: Chlorophyta; C: Mamiellophyceae; O: Mamiellales; F: Mamiellaceae | 40% | - | - | - | - | - | - | - | - |
| P: Chlorophyta; C: Nephroselmidophyceae; O: Nephroselmidales; F: Nephroselmidaceae | 20% | - | 40% | 14% | - | - | - | - | - |
| P: Chlorophyta; C: Palmophyllophyceae; O: Prasinococcales; F: Prasinococcaceae; S: *Prasinococcus capsulatus* | 40% | 17% | 80% | 86% | 50% | - | 14% | - | - |
| P: Chlorophyta; C: Palmophyllophyceae; O: Prasinococcales; F: Prasinococcaceae; G: *Prasinococcus* | 20% | - | 60% | 71% | 50% | 20% | - | - | - |
| P: Chlorophyta; C: Palmophyllophyceae; O: Prasinococcales; F: Prasinococcaceae; S: *Prasinoderma coloniale* | 60% | - | 20% | - | - | - | - | - | - |
| P: Chlorophyta; C: Palmophyllophyceae; O: Prasinococcales; F: Prasinococcaceae | 20% | - | 40% | 29% | 100% | - | - | - | - |
| P: Chlorophyta; C: Palmophyllophyceae; O: Prasinococcales | 80% | - | 100% | 100% | 50% | - | 14% | - | - |
| P: Chlorophyta; C: Picocystophyceae; O: Picocystales | 20% | - | - | - | 50% | - | - | - | - |
| P: Chlorophyta; C: Pyramimonadophyceae; O: Pyramimonadales; F: Pyramimonadaceae; G: *Pyramimonas* | - | 33% | - | 43% | - | - | - | - | - |
| P: Chlorophyta; C: Pyramimonadophyceae; O: Pyramimonadales | 20% | - | 20% | - | 50% | - | - | - | - |
| P: Chlorophyta; C: Trebouxiophyceae; O: Microthamniales | 20% | - | - | - | - | - | - | - | - |
| P: Chlorophyta; C: Ulvophyceae; O: Bryopsidales; F: Bryopsidaceae | 40% | - | 20% | - | - | - | - | - | - |
| P: Chlorophyta; C: Ulvophyceae; O: Bryopsidales; F: Ostreobiaceae; S: *Ostreobium quekettii* | 20% | - | - | - | - | - | - | - | - |
| P: Chlorophyta; C: Ulvophyceae; O: Bryopsidales; F: Ostreobiaceae; G: *Ostreobium* | 20% | - | - | - | - | - | - | - | - |
| P: Chlorophyta; C: Ulvophyceae; O: Bryopsidales; F: Ostreobiaceae | 40% | - | - | - | - | - | - | - | - |
| P: Chlorophyta; C: Ulvophyceae; O: Bryopsidales | 60% | 17% | 20% | 14% | - | - | 14% | - | - |
| P: Chlorophyta; C: Ulvophyceae; O: Cladophorales; F: Cladophoraceae; S: *Cladophora socialis* | 20% | 17% | - | - | - | - | - | - | - |
| P: Chlorophyta; C: Ulvophyceae; O: Cladophorales; F: Cladophoraceae | 20% | - | - | - | - | - | - | - | - |
| P: Chlorophyta; C: Ulvophyceae; O: Cladophorales | 20% | - | - | - | 50% | - | - | - | - |
| P: Chlorophyta; C: Ulvophyceae; O: Ulvales; F: Ulvaceae; G: *Ulva* | 40% | 17% | - | - | - | - | - | - | - |
| P: Chlorophyta; C: Ulvophyceae; O: Ulvales; F: Ulvaceae | 40% | - | 20% | - | 50% | - | 29% | - | - |
| P: Chlorophyta; C: Ulvophyceae; O: Ulvales; F: Ulvellaceae; S: *Ulvella endozoica* | - | 17% | - | 14% | - | - | - | - | - |
| P: Chlorophyta; C: Ulvophyceae; O: Ulvales | 40% | - | - | - | 50% | - | - | - | - |
| P: Chlorophyta; C: Ulvophyceae | - | 17% | 20% | - | - | - | - | - | - |
| P: Chlorophyta; F: Pycnococcaceae | 80% | 17% | - | 14% | - | - | - | - | - |
| P: Chlorophyta | 100% | 33% | 80% | 100% | 100% | 20% | 43% | - | 25% |
| P: Rhodophyta; C: Bangiophyceae; O: Bangiales | 80% | - | 40% | 14% | 50% | - | 29% | - | - |
| P: Rhodophyta; C: Bangiophyceae; O: Cyanidiales | 20% | - | - | - | - | - | - | - | - |
| P: Rhodophyta; C: Compsopogonophyceae; O: Erythropeltidales; F: Erythrotrichiaceae; G: *Erythrotrichia* | 20% | 17% | 20% | 14% | - | - | 29% | - | - |
| P: Rhodophyta; C: Compsopogonophyceae; O: Erythropeltidales; F: Erythrotrichiaceae | 40% | - | 20% | 43% | - | 40% | 71% | - | - |
| P: Rhodophyta; C: Compsopogonophyceae; O: Erythropeltidales | 20% | - | - | - | 50% | - | 43% | - | - |
| P: Rhodophyta; C: Compsopogonophyceae; O: Rhodochaetales; F: Rhodochaetaceae | 20% | - | - | - | - | - | 29% | - | - |
| P: Rhodophyta; C: Compsopogonophyceae; O: Rhodochaetales | 40% | - | - | - | 100% | - | 29% | - | - |
| P: Rhodophyta; C: Florideophyceae; O: Acrochaetiales | 40% | 33% | - | 14% | - | 20% | 14% | - | - |
| P: Rhodophyta; C: Florideophyceae; O: Ahnfeltiales | 80% | 17% | 40% | 43% | - | - | 57% | - | - |
| P: Rhodophyta; C: Florideophyceae; O: Balbianiales; F: Balbianiaceae | 20% | - | - | - | - | - | 14% | - | - |
| P: Rhodophyta; C: Florideophyceae; O: Balbianiales | 40% | 17% | - | - | - | - | 29% | - | - |
| P: Rhodophyta; C: Florideophyceae; O: Batrachospermales | 20% | - | - | - | - | - | 14% | - | - |
| P: Rhodophyta; C: Florideophyceae; O: Bonnemaisoniales; F: Bonnemaisoniaceae | 40% | - | - | - | - | - | 14% | - | - |
| P: Rhodophyta; C: Florideophyceae; O: Bonnemaisoniales; F: Naccariaceae | 40% | - | - | - | - | - | - | - | - |
| P: Rhodophyta; C: Florideophyceae; O: Bonnemaisoniales | 40% | - | - | 14% | 50% | - | 29% | - | - |
| P: Rhodophyta; C: Florideophyceae; O: Ceramiales; F: Callithamniaceae | - | 17% | - | - | - | - | - | - | - |
| P: Rhodophyta; C: Florideophyceae; O: Ceramiales; F: Ceramiaceae; G: *Centroceras* | 20% | - | - | 14% | - | - | - | - | - |
| P: Rhodophyta; C: Florideophyceae; O: Ceramiales; F: Ceramiaceae | 60% | - | 20% | - | 50% | - | 29% | - | - |
| P: Rhodophyta; C: Florideophyceae; O: Ceramiales; F: Dasyaceae; S: *Eupogodon planus* | 20% | - | - | - | - | - | - | - | - |
| P: Rhodophyta; C: Florideophyceae; O: Ceramiales; F: Dasyaceae | 20% | 17% | - | - | 50% | - | - | - | - |
| P: Rhodophyta; C: Florideophyceae; O: Ceramiales; F: Delesseriaceae; S: *Taenioma perpusillum* | 20% | - | - | 14% | - | - | - | - | - |
| P: Rhodophyta; C: Florideophyceae; O: Ceramiales; F: Delesseriaceae; G: *Taenioma* | 20% | - | - | - | - | - | - | - | - |
| P: Rhodophyta; C: Florideophyceae; O: Ceramiales; F: Delesseriaceae | 40% | - | - | - | - | - | - | - | - |
| P: Rhodophyta; C: Florideophyceae; O: Ceramiales; F: Rhodomelaceae; S: *Polysiphonia anomala* | - | 17% | - | 14% | - | - | - | - | - |
| P: Rhodophyta; C: Florideophyceae; O: Ceramiales; F: Rhodomelaceae; S: *Vertebrata aterrima* | 20% | - | - | - | - | - | - | - | - |
| P: Rhodophyta; C: Florideophyceae; O: Ceramiales; F: Rhodomelaceae | 20% | - | 40% | 14% | - | - | 14% | - | - |
| P: Rhodophyta; C: Florideophyceae; O: Ceramiales | 80% | 17% | 20% | 14% | 50% | - | 29% | - | - |
| P: Rhodophyta; C: Florideophyceae; O: Corallinales; F: Corallinaceae; G: *Corallina* | 40% | - | - | 29% | 50% | 20% | 43% | - | - |
| P: Rhodophyta; C: Florideophyceae; O: Corallinales; F: Corallinaceae; G: *Jania* | 20% | - | - | - | - | - | 29% | - | - |
| P: Rhodophyta; C: Florideophyceae; O: Corallinales; F: Corallinaceae; S: *Neogoniolithon megalocystum* | 20% | - | - | - | - | - | - | - | - |
| P: Rhodophyta; C: Florideophyceae; O: Corallinales; F: Corallinaceae | 60% | 50% | 40% | 86% | 100% | 40% | 71% | 100% | - |
| P: Rhodophyta; C: Florideophyceae; O: Corallinales; F: Porolithaceae | 20% | - | - | - | - | - | 29% | - | - |
| P: Rhodophyta; C: Florideophyceae; O: Corallinales | 100% | 17% | 60% | 86% | 100% | 40% | 71% | - | - |
| P: Rhodophyta; C: Florideophyceae; O: Gelidiales; F: Gelidiellaceae; G: *Gelidiella* | - | 17% | - | 14% | - | - | - | - | - |
| P: Rhodophyta; C: Florideophyceae; O: Gigartinales; F: Dicranemataceae | 20% | - | - | - | - | - | - | - | - |
| P: Rhodophyta; C: Florideophyceae; O: Gigartinales; F: Dumontiaceae; S: *Gibsmithia dotyi* | 20% | - | - | - | - | - | - | - | - |
| P: Rhodophyta; C: Florideophyceae; O: Gigartinales; F: Dumontiaceae | 20% | - | - | - | - | - | - | - | - |
| P: Rhodophyta; C: Florideophyceae; O: Gigartinales; F: Gigartinaceae | 20% | - | - | - | - | - | - | - | - |
| P: Rhodophyta; C: Florideophyceae; O: Gigartinales; F: Kallymeniaceae; G: *Leiomenia* | 20% | - | - | - | - | - | 14% | - | - |
| P: Rhodophyta; C: Florideophyceae; O: Gigartinales; F: Kallymeniaceae; S: *Meredithia crenata* | 20% | - | - | - | 100% | - | 14% | - | - |
| P: Rhodophyta; C: Florideophyceae; O: Gigartinales; F: Kallymeniaceae | 40% | - | - | - | 50% | - | 43% | - | - |
| P: Rhodophyta; C: Florideophyceae; O: Gigartinales; F: Peyssonneliaceae; G: *Peyssonnelia* | 20% | - | - | - | - | - | - | - | - |
| P: Rhodophyta; C: Florideophyceae; O: Gigartinales; F: Peyssonneliaceae | 60% | - | 40% | - | 100% | - | 29% | - | - |
| P: Rhodophyta; C: Florideophyceae; O: Gigartinales | 40% | - | 40% | 14% | 50% | 20% | 29% | - | - |
| P: Rhodophyta; C: Florideophyceae; O: Gracilariales | 20% | - | - | - | - | - | 14% | - | - |
| P: Rhodophyta; C: Florideophyceae; O: Halymeniales; F: Halymeniaceae | 20% | - | - | - | 50% | - | 14% | - | - |
| P: Rhodophyta; C: Florideophyceae; O: Halymeniales | 20% | - | - | - | 50% | - | 43% | - | - |
| P: Rhodophyta; C: Florideophyceae; O: Hapalidiales | 20% | - | - | 29% | - | - | - | - | - |
| P: Rhodophyta; C: Florideophyceae; O: Hildenbrandiales; F: Hildenbrandiaceae | 20% | - | - | 29% | 50% | - | 14% | - | - |
| P: Rhodophyta; C: Florideophyceae; O: Nemaliales; F: Liagoraceae | 40% | - | - | - | 50% | - | 14% | - | - |
| P: Rhodophyta; C: Florideophyceae; O: Nemaliales | 40% | - | - | 14% | - | - | 29% | - | - |
| P: Rhodophyta; C: Florideophyceae; O: Nemastomatales | 20% | - | - | - | - | - | 29% | - | - |
| P: Rhodophyta; C: Florideophyceae; O: Plocamiales | 20% | - | - | - | 50% | - | 14% | - | - |
| P: Rhodophyta; C: Florideophyceae; O: Rhodogorgonales | 20% | - | - | - | - | - | - | - | - |
| P: Rhodophyta; C: Florideophyceae; O: Rhodymeniales; F: Champiaceae; S: *Champia hasselbringii* | - | 17% | - | 14% | - | - | - | - | - |
| P: Rhodophyta; C: Florideophyceae; O: Rhodymeniales; F: Champiaceae | 20% | - | - | - | 50% | - | - | - | - |
| P: Rhodophyta; C: Florideophyceae; O: Rhodymeniales; F: Rhodymeniaceae | 40% | - | - | - | - | - | 29% | - | - |
| P: Rhodophyta; C: Florideophyceae; O: Rhodymeniales | 80% | 17% | 20% | 71% | 100% | 40% | 57% | - | - |
| P: Rhodophyta; C: Florideophyceae; O: Sporolithales; F: Sporolithaceae; G: *Sporolithon* | 20% | - | - | 14% | - | - | - | - | - |
| P: Rhodophyta; C: Florideophyceae; O: Sporolithales; F: Sporolithaceae | 20% | - | - | - | 100% | - | - | - | - |
| P: Rhodophyta; C: Florideophyceae; O: Thoreales | 40% | - | - | - | - | - | 14% | - | - |
| P: Rhodophyta; C: Florideophyceae | 60% | 33% | 40% | 86% | 100% | 60% | 43% | - | - |
| P: Rhodophyta | 100% | 83% | 80% | 100% | 100% | 60% | 100% | - | 25% |
| P: Streptophyta; C: Magnoliopsida; O: Alismatales | 20% | - | - | - | - | - | - | - | - |
| P: Streptophyta; C: Magnoliopsida; O: Brassicales; F: Brassicaceae | 20% | - | - | - | - | - | - | 100% | - |
| P: Streptophyta; C: Magnoliopsida | 20% | 33% | 20% | 43% | - | 40% | 29% | - | 100% |
| P: Streptophyta | 20% | - | - | 14% | - | 20% | 29% | - | - |
| C: Chrysophyceae; O: Chromulinales; F: Chromulinaceae | 40% | - | - | - | - | - | - | - | - |
| C: Chrysophyceae; O: Chromulinales | 40% | - | 40% | 57% | - | - | - | - | - |
| C: Dictyochophyceae; O: Rhizochromulinales | 60% | - | 60% | 14% | - | - | - | - | - |
| C: Dinophyceae; O: Dinophysiales; F: Dinophysiaceae; S: *Sinophysis microcephala* | - | 17% | - | - | - | - | - | - | - |
| C: Dinophyceae; O: Gonyaulacales; F: Goniodomataceae; S: *Gambierdiscus belizeanus* | 20% | - | - | - | - | - | - | - | - |
| C: Dinophyceae; O: Gonyaulacales; F: Goniodomataceae; S: *Gambierdiscus carpenteri* | 20% | - | 20% | - | - | - | - | - | - |
| C: Dinophyceae; O: Gonyaulacales; F: Goniodomataceae; G: *Gambierdiscus* | 20% | 33% | 40% | - | - | - | - | - | - |
| C: Dinophyceae; O: Gonyaulacales; F: Pyrophacaceae; G: *Fragilidium* | 20% | - | 40% | 14% | - | 20% | - | - | - |
| C: Dinophyceae; O: Gonyaulacales | 80% | 17% | 20% | 14% | 50% | 20% | 14% | - | - |
| C: Dinophyceae; O: Gymnodiniales; F: Gymnodiniaceae; G: *Amphidinium* | 60% | 33% | 100% | 100% | - | 80% | 43% | - | - |
| C: Dinophyceae; O: Gymnodiniales; F: Gymnodiniaceae; S: *Ankistrodinium semilunatum* | - | 17% | - | - | - | - | - | - | - |
| C: Dinophyceae; O: Gymnodiniales; F: Gymnodiniaceae; G: *Ankistrodinium* | - | 17% | - | - | - | - | - | - | - |
| C: Dinophyceae; O: Gymnodiniales; F: Gymnodiniaceae; G: *Gymnodinium* | 20% | - | - | 14% | - | - | - | - | - |
| C: Dinophyceae; O: Gymnodiniales; F: Gymnodiniaceae; G: *Moestrupia* | 40% | - | 20% | 14% | - | - | - | - | - |
| C: Dinophyceae; O: Gymnodiniales; F: Gymnodiniaceae; G: *Paragymnodinium* | 20% | - | 20% | 43% | - | - | - | - | - |
| C: Dinophyceae; O: Gymnodiniales; F: Gymnodiniaceae; S: *Testudodinium corrugatum* | - | 17% | - | - | - | - | - | - | - |
| C: Dinophyceae; O: Gymnodiniales; F: Gymnodiniaceae | 80% | 33% | 80% | 71% | 100% | - | 14% | - | - |
| C: Dinophyceae; O: Gymnodiniales | 60% | 17% | 80% | - | 50% | - | 29% | - | - |
| C: Dinophyceae; O: Peridiniales; F: Kryptoperidiniaceae; S: *Durinskia baltica* | 20% | - | - | - | - | - | - | - | - |
| C: Dinophyceae; O: Peridiniales; F: Kryptoperidiniaceae; G: *Durinskia* | 20% | 50% | 40% | - | - | 20% | - | - | - |
| C: Dinophyceae; O: Peridiniales; F: Kryptoperidiniaceae; S: *Kryptoperidinium foliaceum* | 20% | - | - | - | - | - | - | - | - |
| C: Dinophyceae; O: Peridiniales; F: Kryptoperidiniaceae; G: *Kryptoperidinium* | 20% | - | - | - | - | - | - | - | - |
| C: Dinophyceae; O: Peridiniales; F: Kryptoperidiniaceae | 40% | - | - | - | - | - | 14% | - | - |
| C: Dinophyceae; O: Peridiniales; F: Ostreopsidaceae; S: *Coolia canariensis* | 20% | - | 20% | - | - | - | - | - | - |
| C: Dinophyceae; O: Peridiniales; F: Ostreopsidaceae; G: *Ostreopsis* | 40% | 17% | 60% | - | - | 20% | 14% | - | - |
| C: Dinophyceae; O: Peridiniales; F: Ostreopsidaceae | 40% | 17% | - | 14% | - | - | - | - | - |
| C: Dinophyceae; O: Peridiniales; F: Peridiniaceae; S: *Bysmatrum subsalsum* | 60% | 33% | 100% | 71% | - | 40% | - | - | - |
| C: Dinophyceae; O: Peridiniales; F: Peridiniaceae; G: *Bysmatrum* | 40% | 17% | 80% | 14% | - | 20% | - | - | - |
| C: Dinophyceae; O: Peridiniales; F: Peridiniaceae; S: *Peridinium sociale* | 20% | - | - | - | - | - | - | - | - |
| C: Dinophyceae; O: Peridiniales; F: Peridiniaceae | 40% | - | - | 29% | 100% | 20% | - | - | - |
| C: Dinophyceae; O: Peridiniales; F: Podolampaceae; G: *Lessardia* | 20% | - | - | - | - | - | - | - | - |
| C: Dinophyceae; O: Peridiniales; F: Podolampaceae; G: *Podolampas* | 20% | - | - | - | - | - | - | - | - |
| C: Dinophyceae; O: Peridiniales; S: *Caladoa arcachonensis* | 40% | - | 40% | 14% | - | - | - | - | - |
| C: Dinophyceae; O: Peridiniales | 80% | 17% | 100% | 57% | 50% | 40% | 43% | - | - |
| C: Dinophyceae; O: Prorocentrales; F: Prorocentraceae; S: *Prorocentrum concavum* | - | 17% | - | - | - | - | - | - | - |
| C: Dinophyceae; O: Prorocentrales; F: Prorocentraceae; S: *Prorocentrum lima* | 20% | - | - | - | - | - | - | - | - |
| C: Dinophyceae; O: Prorocentrales; F: Prorocentraceae; G: *Prorocentrum* | - | 17% | 40% | 43% | - | 20% | - | - | - |
| C: Dinophyceae; O: Prorocentrales; F: Prorocentraceae | 60% | - | - | 14% | 50% | 20% | - | - | - |
| C: Dinophyceae; O: Prorocentrales | 100% | - | 20% | - | 50% | - | 14% | - | - |
| C: Dinophyceae; O: Pyrocystales; F: Pyrocystaceae | 20% | - | - | - | - | - | - | - | - |
| C: Dinophyceae; O: Pyrocystales | 40% | - | 20% | - | - | - | - | - | - |
| C: Dinophyceae; O: Suessiales; F: Symbiodiniaceae; G: *Symbiodinium* | 40% | 50% | 100% | 100% | - | 60% | 43% | - | - |
| C: Dinophyceae; O: Suessiales; F: Symbiodiniaceae | 100% | 17% | 60% | 57% | 100% | - | 29% | - | 50% |
| C: Dinophyceae; O: Suessiales | 40% | - | 20% | - | 100% | - | 14% | - | 50% |
| C: Dinophyceae; O: Syndiniales; F: Amoebophryaceae; G: *Amoebophrya* | - | 17% | 20% | - | - | - | - | - | - |
| C: Dinophyceae; O: Thoracosphaerales; F: Thoracosphaeraceae; S: *Pachena abriliae* | - | 17% | - | - | - | - | - | - | - |
| C: Dinophyceae; O: Thoracosphaerales; F: Thoracosphaeraceae | 20% | - | - | - | 100% | - | - | - | 25% |
| C: Dinophyceae; O: Thoracosphaerales | 20% | - | - | - | 50% | - | - | - | - |
| C: Dinophyceae; S: *Pyramidodinium atrofuscum* | 20% | - | 20% | - | - | 20% | - | - | - |
| C: Dinophyceae; G: *Pyramidodinium* | - | 17% | - | - | - | - | - | - | - |
| C: Dinophyceae | 100% | 50% | 100% | 100% | 100% | 80% | 43% | 100% | 50% |
| C: Pelagophyceae; O: Pelagomonadales; S: *Aureoumbra lagunensis* | 20% | - | - | - | - | - | - | - | - |
| C: Pelagophyceae; O: Pelagomonadales; G: *Aureoumbra* | 20% | - | - | - | - | - | - | - | - |
| C: Pelagophyceae; O: Pelagomonadales | 40% | - | - | - | 50% | - | 14% | - | - |
| C: Pelagophyceae; O: Sarcinochrysidales; F: Chrysocystaceae | 40% | - | - | - | - | - | - | - | - |
| C: Pelagophyceae; O: Sarcinochrysidales; F: Sarcinochrysidaceae;  S: *Glomerochrysis psammophila* | - | 17% | 40% | 57% | - | - | - | - | - |
| C: Pelagophyceae; O: Sarcinochrysidales; F: Sarcinochrysidaceae | 20% | - | 20% | 14% | 50% | - | - | - | - |
| C: Pelagophyceae; O: Sarcinochrysidales | 80% | - | 20% | 14% | - | - | - | - | - |
| C: Pelagophyceae | 100% | - | 20% | 57% | 50% | - | - | - | - |
| C: Phaeophyceae; O: Dictyotales; F: Dictyotaceae; G: *Dictyopteris* | 20% | - | - | - | - | - | 14% | - | - |
| C: Phaeophyceae; O: Dictyotales; F: Dictyotaceae; S: *Lobophora declerckii* | 40% | - | - | - | - | 20% | - | - | - |
| C: Phaeophyceae; O: Dictyotales; F: Dictyotaceae; G: *Lobophora* | 80% | 17% | - | 43% | 50% | 40% | 43% | - | 25% |
| C: Phaeophyceae; O: Dictyotales; F: Dictyotaceae | 20% | - | 20% | 14% | 50% | - | 29% | - | - |
| C: Phaeophyceae; O: Dictyotales | 40% | - | - | - | 50% | - | 43% | - | - |
| C: Phaeophyceae; O: Ectocarpales; F: Chordariaceae | - | 17% | - | 29% | - | 20% | - | - | - |
| C: Phaeophyceae; O: Ectocarpales; F: Ectocarpaceae | 20% | - | - | - | - | - | - | - | - |
| C: Phaeophyceae; O: Ectocarpales; F: Scytosiphonaceae | 40% | - | - | - | - | - | - | - | - |
| C: Phaeophyceae; O: Ectocarpales | 60% | 17% | 40% | 29% | 100% | - | 29% | - | - |
| C: Phaeophyceae; O: Fucales; F: Sargassaceae; G: *Sargassum* | - | 33% | - | - | 50% | - | 14% | - | - |
| C: Phaeophyceae; O: Fucales; F: Sargassaceae | 40% | - | - | - | 50% | - | 14% | - | - |
| C: Phaeophyceae; O: Fucales | 60% | 17% | 80% | 57% | 50% | 40% | 57% | - | - |
| C: Phaeophyceae; O: Laminariales | 40% | 17% | - | 14% | 50% | - | 43% | - | - |
| C: Phaeophyceae | 40% | - | 20% | 43% | 50% | 20% | 29% | - | - |
| C: Pinguiophyceae; O: Pinguiochrysidales; F: Pinguiochrysidaceae; G: *Polypodochrysis* | 40% | - | - | - | - | - | - | - | - |
| C: Pinguiophyceae; O: Pinguiochrysidales | 20% | - | - | - | - | - | - | - | - |
| C: Synchromophyceae | 40% | - | - | 14% | 50% | - | - | - | - |
