# Supplementary material for: Looking for the sponge loop: analyses of detritus on a Caribbean forereef using stable isotope and eDNA metabarcoding techniques
Source: PeerJ. 2024 Feb 23;12:e16970. doi: 10.7717/peerj.16970 (PMC10896084; doi:10.7717/peerj.16970)
Supplement: Table S7 — Classification includes phylum (P), class (C), order (O), family (F), genus (G), and species (S), where applicable. EAM = detritus suctioned from epilithic algal matrix in July 2018 (J18; n = 5) and March 2019 (M19, n = 6). sed. trap = particulates captured in sediment traps in July 2018 (n = 5) and March 2019 (n = 7). tray = detritus sampled from trays placed under reef overhangs in March 2019 (n = 2). h’vore fec = feces collected from herbivorous fishes in 2018 (n = 5) and 2019 (n = 7). s’vore fec = feces collected from spongivore fishes in 2018 (n = 1) and 2019 (n = 4). [file peerj-12-16970-s011.docx]

| Classification | EAM | | sed. trap | | tray | h'vore fec | | s'vore fec | |
| --- | --- | --- | --- | --- | --- | --- | --- | --- | --- |
|  | J18 | M19 | J18 | M19 | M19 | J18 | M19 | J18 | M19 |
| P: Annelida; C: Polychaeta; O: Eunicida; F: Amphinomidae | - | 17% | - | - | - | - | - | - | - |
| P: Annelida; C: Polychaeta; O: Eunicida; F: Dorvilleidae | - | 17% | - | - | - | - | - | - | - |
| P: Annelida; C: Polychaeta; O: Eunicida | - | 17% | - | - | 50% | - | - | - | - |
| P: Annelida; C: Polychaeta; O: Phyllodocida; F: Hesionidae | - | 33% | - | - | - | - | - | - | - |
| P: Annelida; C: Polychaeta; O: Phyllodocida; F: Syllidae; G: *Erinaceusyllis* | - | 17% | - | - | - | - | - | - | - |
| P: Annelida; C: Polychaeta; O: Phyllodocida; F: Syllidae; G: *Salvatoria* | - | 33% | - | - | - | - | - | - | - |
| P: Annelida; C: Polychaeta; O: Phyllodocida; F: Syllidae | - | 33% | - | - | - | - | - | - | - |
| P: Annelida; C: Polychaeta; O: Phyllodocida | 20% | 50% | 20% | - | - | - | 14% | - | - |
| P: Annelida; C: Polychaeta; O: Spionida; F: Spionidae; G: *Laonice* | - | 17% | - | - | - | - | - | - | - |
| P: Annelida; C: Polychaeta; F: Capitellidae | 20% | - | 20% | - | 50% | - | - | - | - |
| P: Annelida; C: Polychaeta; F: Protodrilidae; S: *Megadrilus hochbergi* | - | 17% | - | - | - | - | - | - | - |
| P: Annelida; C: Polychaeta; F: Protodrilidae; G: *Protodrilus* | - | 17% | - | - | - | - | - | - | - |
| P: Annelida; C: Polychaeta | - | 33% | - | 14% | - | - | - | - | - |
| P: Annelida | 20% | 17% | - | 14% | 50% | - | 14% | - | - |
| P: Arthropoda; C: Arachnida; O: Trombidiformes; F: Halacaridae | 40% | - | - | 43% | - | 20% | - | - | - |
| P: Arthropoda; C: Arachnida; O: Trombidiformes | 40% | - | - | - | 50% | - | - | - | - |
| P: Arthropoda; C: Hexanauplia; O: Calanoida; F: Paracalanidae; S: *Paracalanus indicus* | 20% | - | - | - | - | - | - | - | - |
| P: Arthropoda; C: Hexanauplia; O: Calanoida; F: Pseudocyclopidae; S: *Pseudocyclops juanibali* | - | 17% | - | 14% | - | - | - | - | - |
| P: Arthropoda; C: Hexanauplia; O: Calanoida; F: Pseudocyclopidae; G: *Pseudocyclops* | - | 17% | - | 14% | - | - | - | - | - |
| P: Arthropoda; C: Hexanauplia; O: Calanoida; F: Pseudocyclopidae | - | 17% | - | - | 100% | - | - | - | - |
| P: Arthropoda; C: Hexanauplia; O: Calanoida | 20% | 17% | - | 14% | 100% | - | 14% | - | - |
| P: Arthropoda; C: Hexanauplia; O: Cyclopoida; F: Cyclopettidae | - | 17% | - | - | - | - | - | - | - |
| P: Arthropoda; C: Hexanauplia; O: Cyclopoida; F: Cyclopinidae; S: *Cyclopina gracilis* | - | 17% | - | - | - | - | - | - | - |
| P: Arthropoda; C: Hexanauplia; O: Cyclopoida; F: Cyclopinidae; G: *Cyclopina* | 40% | 33% | - | 14% | - | - | - | - | - |
| P: Arthropoda; C: Hexanauplia; O: Cyclopoida; F: Cyclopinidae | 40% | 50% | - | 14% | 50% | - | - | - | - |
| P: Arthropoda; C: Hexanauplia; O: Cyclopoida; F: Schminkepinellidae; G: *Cyclopinella* | - | 17% | - | - | - | - | - | - | - |
| P: Arthropoda; C: Hexanauplia; O: Cyclopoida; F: Smirnovipinidae | - | 17% | - | - | - | - | - | - | - |
| P: Arthropoda; C: Hexanauplia; O: Cyclopoida | 40% | 50% | - | 14% | 50% | - | - | - | - |
| P: Arthropoda; C: Hexanauplia; O: Harpacticoida; F: Ameiridae; G: *Ameira* | 20% | - | 20% | - | - | - | - | - | - |
| P: Arthropoda; C: Hexanauplia; O: Harpacticoida; F: Ameiridae | 40% | - | - | - | 50% | - | - | - | - |
| P: Arthropoda; C: Hexanauplia; O: Harpacticoida; F: Dactylopusiidae | 20% | - | - | - | 50% | - | - | - | - |
| P: Arthropoda; C: Hexanauplia; O: Harpacticoida; F: Louriniidae | 20% | - | - | - | - | - | - | - | - |
| P: Arthropoda; C: Hexanauplia; O: Harpacticoida; F: Miraciidae; G: *Sarsamphiascus* | - | 33% | - | - | - | - | - | - | - |
| P: Arthropoda; C: Hexanauplia; O: Harpacticoida; F: Miraciidae; G: *Typhlamphiascus* | - | 50% | - | - | - | - | - | - | - |
| P: Arthropoda; C: Hexanauplia; O: Harpacticoida; F: Miraciidae | 40% | 33% | 20% | 29% | 50% | - | - | - | - |
| P: Arthropoda; C: Hexanauplia; O: Harpacticoida; F: Normanellidae | 40% | 17% | - | 29% | 50% | - | - | - | - |
| P: Arthropoda; C: Hexanauplia; O: Harpacticoida | 100% | 50% | 20% | 14% | 50% | - | - | - | - |
| P: Arthropoda; C: Hexanauplia | - | 33% | - | 29% | 50% | - | - | - | - |
| P: Arthropoda; C: Ostracoda; O: Myodocopida; F: Cylindroleberididae | 40% | - | - | - | - | - | - | - | - |
| P: Arthropoda; C: Ostracoda; O: Myodocopida; F: Sarsiellidae; G: *Eusarsiella* | - | 17% | - | - | - | - | - | - | - |
| P: Arthropoda; C: Ostracoda; O: Myodocopida; F: Sarsiellidae | - | 17% | - | - | - | - | - | - | - |
| P: Arthropoda; C: Ostracoda; O: Myodocopida | 20% | 17% | - | - | - | - | - | - | - |
| P: Arthropoda; C: Ostracoda; O: Podocopida; F: Bairdiidae | - | 17% | - | - | - | - | - | - | - |
| P: Arthropoda; C: Ostracoda; O: Podocopida; F: Cytheruridae | 20% | - | - | - | - | - | - | - | - |
| P: Arthropoda; C: Ostracoda; O: Podocopida; F: Hemicytheridae; G: *Aurila* | 20% | 17% | - | 29% | - | - | - | - | - |
| P: Arthropoda; C: Ostracoda; O: Podocopida; F: Hemicytheridae | - | 17% | - | 14% | - | - | - | - | - |
| P: Arthropoda; C: Ostracoda; O: Podocopida | 20% | 17% | 60% | 29% | 50% | 20% | - | - | - |
| P: Arthropoda | 80% | 33% | 20% | 43% | 100% | - | 14% | - | - |
| P: Chordata; C: Actinopteri; O: Labriformes; F: Labridae; G: *Sparisoma* | - | 17% | - | - | - | - | - | - | - |
| P: Chordata; C: Actinopteri; O: Labriformes | - | 17% | - | - | - | - | - | - | - |
| P: Chordata; C: Actinopteri | 20% | - | - | - | - | - | - | 100% | 50% |
| P: Chordata; C: Ascidiacea; O: Phlebobranchia; F: Perophoridae | 20% | - | - | - | - | - | - | - | - |
| P: Chordata; C: Mammalia | 20% | 17% | 20% | 43% | - | 40% | 14% | 100% | - |
| P: Chordata | 40% | - | 60% | 29% | 50% | - | - | 100% | 25% |
| P: Cnidaria; C: Anthozoa; O: Alcyonacea; F: Gorgoniidae | 60% | - | 20% | 14% | 50% | - | 14% | - | 75% |
| P: Cnidaria; C: Anthozoa; O: Alcyonacea; F: Plexauridae | 40% | - | - | - | - | - | - | - | 25% |
| P: Cnidaria; C: Anthozoa; O: Alcyonacea | 40% | 17% | 40% | 43% | - | - | 14% | - | 50% |
| P: Cnidaria; C: Anthozoa; O: Scleractinia; F: Pocilloporidae; S: *Stylophora pistillata* | 20% | - | - | - | - | - | - | - | - |
| P: Cnidaria; C: Anthozoa; O: Zoantharia; F: Parazoanthidae | - | 17% | - | - | - | - | - | - | - |
| P: Cnidaria; C: Hydrozoa; O: Anthoathecata | 20% | - | - | - | - | - | - | - | - |
| P: Cnidaria; C: Hydrozoa; O: Leptothecata; F: Aequoreidae; S: *Aequorea australis* | 20% | - | 20% | - | - | - | - | - | - |
| P: Cnidaria; C: Hydrozoa; O: Leptothecata; F: Clytiidae; S: *Clytia hemisphaerica* | 20% | - | - | - | - | - | - | - | - |
| P: Cnidaria; C: Hydrozoa | - | 17% | - | - | - | - | - | - | - |
| P: Cnidaria | 40% | 17% | 60% | 14% | 50% | - | - | - | 75% |
| P: Echinodermata; C: Holothuroidea; O: Apodida; F: Synaptidae; S: *Leptosynapta clarki* | - | 17% | - | - | - | - | - | - | - |
| P: Gastrotricha; O: Chaetonotida; F: Chaetonotidae; S: *Aspidiophorus tentaculatus* | - | 17% | - | - | - | - | - | - | - |
| P: Gastrotricha; O: Macrodasyida; F: Thaumastodermatidae | - | 50% | - | - | - | - | - | - | - |
| P: Gastrotricha; O: Macrodasyida | - | 17% | - | - | 50% | - | - | - | - |
| P: Gnathostomulida; O: Bursovaginoidea; F: Austrognathiidae; S: *Austrognatharia strunki* | - | 17% | - | - | - | - | - | - | - |
| P: Mollusca; C: Bivalvia; O: Adapedonta | - | 33% | - | - | - | - | - | - | - |
| P: Mollusca; C: Gastropoda; O: Cephalaspidea; F: Haminoeidae | 20% | - | - | - | - | - | - | - | - |
| P: Mollusca; C: Gastropoda; O: Cephalaspidea | 20% | - | - | - | - | - | - | - | - |
| P: Mollusca | 80% | 33% | 40% | 43% | 50% | - | 14% | - | 25% |
| P: Nematoda; C: Chromadorea; O: Chromadorida; F: Chromadoridae; G: *Chromadorita* | 20% | 17% | - | - | - | - | - | - | - |
| P: Nematoda; C: Chromadorea; O: Chromadorida; F: Chromadoridae | 20% | 50% | 40% | 14% | - | - | - | - | - |
| P: Nematoda; C: Chromadorea; O: Chromadorida | 40% | 17% | - | - | 50% | 20% | - | - | - |
| P: Nematoda; C: Chromadorea; O: Desmodorida; F: Desmodoridae; S: *Laxus oneistus* | - | 17% | - | - | - | - | - | - | - |
| P: Nematoda; C: Chromadorea; O: Desmodorida; F: Desmodoridae; G: *Robbea* | - | 17% | - | - | - | - | - | - | - |
| P: Nematoda; C: Chromadorea; O: Desmodorida; F: Desmodoridae | - | 17% | - | - | - | - | - | - | - |
| P: Nematoda; C: Chromadorea; O: Desmodorida | 20% | 17% | - | - | - | - | - | - | - |
| P: Nematoda; C: Chromadorea; O: Desmoscolecida | 20% | - | - | - | - | - | - | - | - |
| P: Nematoda; C: Enoplea; O: Enoplida; F: Oncholaimidae; G: *Meyersia* | - | 17% | - | - | - | - | - | - | - |
| P: Nematoda | 60% | - | - | 14% | 100% | - | - | - | - |
| P: Nemertea; C: Enopla; O: Monostilifera | - | 17% | - | - | - | - | - | - | - |
| P: Nemertea; C: Palaeonemertea; F: Cephalothricidae; G: *Cephalothrix* | - | 17% | - | - | - | - | - | - | - |
| P: Nemertea; C: Palaeonemertea | - | 17% | - | - | - | - | - | - | - |
| P: Platyhelminthes; C: Catenulida; F: Catenulidae; G: *Paracatenula* | - | 17% | - | - | - | - | - | - | - |
| P: Platyhelminthes; C: Catenulida; F: Catenulidae | - | 17% | - | - | - | - | - | - | - |
| P: Platyhelminthes; C: Rhabditophora; O: Macrostomida; F: Dolichomacrostomidae; S: *Myozonaria bistylifera* | - | 33% | - | - | - | - | - | - | - |
| P: Platyhelminthes; C: Rhabditophora; O: Macrostomida; F: Dolichomacrostomidae | - | 33% | - | - | - | - | - | - | - |
| P: Platyhelminthes; C: Rhabditophora; O: Macrostomida; F: Macrostomidae | 40% | - | - | - | - | - | - | - | - |
| P: Platyhelminthes; C: Rhabditophora; O: Macrostomida; F: Microstomidae; G: *Microstomum* | - | 33% | - | - | - | - | - | - | - |
| P: Platyhelminthes; C: Rhabditophora; O: Macrostomida | 40% | - | - | - | 50% | - | - | - | - |
| P: Platyhelminthes; C: Rhabditophora; O: Proseriata; F: Otoplanidae | - | 33% | - | 14% | - | - | - | - | - |
| P: Platyhelminthes; C: Rhabditophora; O: Proseriata | - | 17% | - | - | - | - | - | - | - |
| P: Platyhelminthes; C: Rhabditophora; O: Rhabdocoela | - | 33% | - | - | - | - | - | - | - |
| P: Platyhelminthes; C: Trematoda; O: Azygiida | 20% | - | - | - | - | - | - | - | - |
| P: Platyhelminthes | 40% | 33% | - | - | 50% | - | - | - | 50% |
| P: Rotifera; C: Monogononta; O: Ploima; F: Trichocercidae; G: *Trichocerca* | - | 17% | - | - | - | - | - | - | - |
| P: Rotifera; C: Monogononta; O: Ploima | 20% | - | - | - | - | - | - | - | - |
| P: Rotifera | 20% | 17% | 40% | - | - | 20% | - | - | - |
| P: Sipuncula; O: Aspidosiphonidormes; F: Aspidosiphonidae | 20% | - | - | - | - | - | - | - | - |
| P: Sipuncula; O: Aspidosiphonidormes | 20% | - | - | - | - | - | - | - | - |
| P: Sipuncula | 20% | - | - | - | - | - | - | - | - |
| P: unspecified metazoa | 16% | 11% | - | - | 25% | - | - | - | - |
| P: Xenacoelomorpha; O: Acoela; F: Convolutidae | 20% | - | - | - | - | - | - | - | - |
| P: Xenacoelomorpha; O: Acoela; F: Haploposthiidae; S: *Kuma albiventer* | - | 17% | - | - | - | - | - | - | - |
| P: Xenacoelomorpha; O: Acoela; F: Haploposthiidae | - | 33% | - | - | - | - | - | - | - |
| P: Xenacoelomorpha; O: Acoela; F: Isodiametridae; S: *Avagina marci* | - | 33% | - | - | - | - | - | - | - |
| P: Xenacoelomorpha; O: Acoela; F: Isodiametridae; G: *Proaphanostoma* | - | 17% | - | - | - | - | - | - | - |
| P: Xenacoelomorpha; O: Acoela; F: Isodiametridae | - | 50% | - | - | - | - | - | - | - |
| P: Xenacoelomorpha; O: Acoela | 80% | 67% | - | - | 50% | - | - | - | - |
| P: Xenacoelomorpha | 20% | 33% | - | - | 100% | - | - | - | - |
