# Supplementary material for: Looking for the sponge loop: analyses of detritus on a Caribbean forereef using stable isotope and eDNA metabarcoding techniques
Source: PeerJ. 2024 Feb 23;12:e16970. doi: 10.7717/peerj.16970 (PMC10896084; doi:10.7717/peerj.16970)
Supplement: Data S1 — Raw stable isotope measurements are available in SIAdata_30sept2023.xlsx, and code for SIA analyses are available in files starting with “SIMMR_*.Rmd”. The raw sequence tables from metabarcoding are available in .csv files starting with “edna_CO1”, “edna_18S”, and “edna_16S”, and the code to analyze sequence data are in .Rmd files starting with “edna_1_analysis”, “edna_2_analysis”, and “edna_3_analysis”. The pdfs in “markdown_outputs/” subdirectory are retained for quick reference and reproducible by knitting the .Rmds. [file peerj-12-16970-s012.zip › data_peerj/markdown_outputs/edna_1_analysis_dataprep_30sept.pdf]

dataprep

Lauren Olinger

9/30/2023

Loading Necessary Libraries

```
library(readr)
```

```
## Warning: package 'readr' was built under R version 4.1.2
```

```
library(plyr)
```

```
## Warning: package 'plyr' was built under R version 4.1.2
```

```
library(vegan)
```

```
## Warning: package 'vegan' was built under R version 4.1.2

## Loading required package: permute

## Warning: package 'permute' was built under R version 4.1.2

## Loading required package: lattice

## This is vegan 2.6-2
```

```
library(ggplot2)
library(pairwiseAdonis)
```

```
## Loading required package: cluster
```

```
library(dplyr)
```

```
## Warning: package 'dplyr' was built under R version 4.1.2

##
## Attaching package: 'dplyr'

## The following objects are masked from 'package:plyr':
##
##     arrange, count, desc, failwith, id, mutate, rename, summarise,
##     summarize

## The following objects are masked from 'package:stats':
##
##     filter, lag

## The following objects are masked from 'package:base':
##
##     intersect, setdiff, setequal, union
```

Data Import

We are importing three CSV files, each corresponding to a different genetic marker (`16S`, `18S`, and `CO1`). Another dataset (`origdeltadat`) containing sample information is also imported.

```
s16s <- read_csv("input/16S ESV Taxonomy Sequence Table (no tissue).csv")
```

```
## Rows: 33820 Columns: 49
## -- Column specification --------------------------------------------------------
## Delimiter: ","
## chr  (9): hash, sequence, kingdom, phylum, class, order, family, genus, species
## dbl (40): ST 1-18, ST 2-18, ST 3-18, ST 4-18, ST 5-18, SS 1-18, SS 2-18, SS ...
##
## i Use `spec()` to retrieve the full column specification for this data.
## i Specify the column types or set `show_col_types = FALSE` to quiet this message.
```

```
s18s <- read_csv("input/18S ESV Taxonomy Sequence Table (no tissue).csv")
```

```
## Rows: 18640 Columns: 51
## -- Column specification --------------------------------------------------------
## Delimiter: ","
## chr  (9): hash, sequence, kingdom, phylum, class, order, family, genus, species
## dbl (42): ST 1-18, ST 2-18, ST 3-18, ST 4-18, ST 5-18, SS 1-18, SS 2-18, SS ...
##
## i Use `spec()` to retrieve the full column specification for this data.
## i Specify the column types or set `show_col_types = FALSE` to quiet this message.
```

```
sCOI <- read_csv("input/C01 ESV Taxonomy Sequence Table (no tissue).csv")
```

```
## Rows: 3126 Columns: 49
## -- Column specification --------------------------------------------------------
## Delimiter: ","
## chr  (9): hash, sequence, kingdom, phylum, class, order, family, genus, species
## dbl (40): ST 1-18, ST 2-18, ST 3-18, ST 4-18, ST 5-18, SS 1-18, SS 2-18, SS ...
##
## i Use `spec()` to retrieve the full column specification for this data.
## i Specify the column types or set `show_col_types = FALSE` to quiet this message.
```

```
origdeltadat <- read_csv("input/sampleinfodelta_4july.csv")
```

```
## Rows: 186 Columns: 28
## -- Column specification --------------------------------------------------------
## Delimiter: ","
## chr (19): category, sample type, species, spp code, samplespp, sample note, ...
## dbl  (9): Index, Year, Sample Mass (mg), sample mass (mg) acidified, Carbon ...
##
## i Use `spec()` to retrieve the full column specification for this data.
## i Specify the column types or set `show_col_types = FALSE` to quiet this message.
```

Data Manipulation

We add a new column named "marker" to each of the three genetic marker dataframes to identify the source of the data. The three datasets are then combined into one dataframe (`seqdat_all`). To achieve this, the columns of each dataset are reordered to match a common order, after which the datasets are concatenated.

```
#make new dataframe "seqdat_all" that combines the top three markers, also adding a column for "marker"
s16s <- cbind(marker = rep('16s', nrow(s16s)), s16s)
s18s <- cbind(marker = rep('18s', nrow(s18s)), s18s)
```

```r
sCOI <- cbind(marker = rep("COI", nrow(sCOI)), sCOI)

#column list from the three csvs (they dont match up across the markers so have to xref)
newcols <- unique(c(colnames(s16s), colnames(sCOI), colnames(s18s)))

#preallocate the dataframe & set colnames
seqdat_all <-
  data.frame(matrix(
    ncol = length(newcols),
    nrow = c(nrow(s16s) + nrow(s18s) + nrow(sCOI))
  ))
colnames(seqdat_all) <- newcols

#reordering colnames of seqdat, s16s, s18s, sCOI, so they match up
reorddat<- function(colname,dfname){
  dfname2 <- dfname[,1:which(colnames(dfname) == colname)]
  dfnametoReord <- dfname[,c((which(colnames(dfname) == colname)+1):ncol(dfname))]
  dfnametoReord <- dfnametoReord[,order(colnames(dfnametoReord))]
  dfname3 <- cbind(dfname2, dfnametoReord)
  return(dfname3)
}

seqdat_all <- reorddat("species",seqdat_all)
s16s <- reorddat("species",s16s)
s18s <- reorddat("species",s18s)
sCOI <- reorddat("species",sCOI)

#populate seqdat_all
seqdat_all[1:nrow(sCOI), which(colnames(seqdat_all) %in%  colnames(sCOI))] <-
  sCOI
seqdat_all[(nrow(sCOI) + 1):(nrow(sCOI) + nrow(s16s)), which(colnames(seqdat_all) %in%  colnames(s16s))]
  s16s
seqdat_all[(nrow(sCOI) + nrow(s16s) + 1):(nrow(seqdat_all)), which(colnames(seqdat_all) %in%  colnames(s
  s18s

seqdat_all[which(is.na(seqdat_all),arr.ind = T)] <- 0
newcols <- colnames(seqdat_all)

# get the info part from seqdat_all so can tack on to subsetted pieces later
seqdat_info <- seqdat_all[,1:which(colnames(seqdat_all) == "species")]

#crossref sample names with the years/ groups/categories from origdeltadat,
#note: deltadat_all includes delta values- this is where could come back and pull those from if want
deltadat_all <-
  origdeltadat[which(origdeltadat$ednacode %in% newcols[grep("-", newcols)]), ]

#remove stuff
rm(s16s, s18s, sCOI, newcols, origdeltadat)
```

Taxonomic Level Summarization

The data is summarized at different taxonomic levels such as kingdom, phylum, class, order, family, genus, and species. The data is further aggregated at the kingdom-phylum (`kp`), kingdom-phylum-class-order (`kpco`),

and species-level resolution.

```
#functions needed to summarize (may not use max)
aggFunctionSum <- function(dataframe, toAverage, toGroup) {
  aggregate(dataframe[, toAverage], dataframe[, toGroup], sum)
}

#sample names for later agg function
seqcolnames <- colnames(seqdat_all)[c((which(colnames(seqdat_all) == "species")+1):ncol(seqdat_all))]

seqdat_all$kingdom[which(seqdat_all$kingdom=="0")]<- "K"
seqdat_all$phylum[which(seqdat_all$phylum=="0")]<- "P"
seqdat_all$class[which(seqdat_all$class=="0")]<- "C"
seqdat_all$order[which(seqdat_all$order=="0")]<- "O"
seqdat_all$family[which(seqdat_all$family=="0")]<- "F"
seqdat_all$genus[which(seqdat_all$genus=="0")]<- "G"
seqdat_all$species[which(seqdat_all$species=="0")]<- "S"

#seqdat_kpcofgs = kingdom/phylum/class/order/fam/genus/species
seqdat_kpcofgs <- cbind(
  kingdom = seqdat_all$kingdom,
  phylum = seqdat_all$phylum,
  class = seqdat_all$class,
  order = seqdat_all$order,
  family = seqdat_all$family,
  genus = seqdat_all$genus,
  species = seqdat_all$species,
  seqdat_all[,c((which(colnames(seqdat_all) == "species")+1):ncol(seqdat_all))]
)

#kp - for nice overview tables
seqdat_kp <- aggFunctionSum(seqdat_kpcofgs, seqcolnames, c("kingdom", "phylum"))

#kpco - for matching to multivariate
seqdat_kpco <- aggFunctionSum(seqdat_kpcofgs, seqcolnames, c("kingdom", "phylum","class","order"))

#seqdat_kpcofgs - for species-level resolution, where species are identified
seqdat_kpcofgs <- aggFunctionSum(seqdat_kpcofgs, seqcolnames, c("kingdom", "phylum","class","order","fa
```

Sample Type Matrix Creation

A matrix containing different sample types and categories, with sample counts (`ns`), is created.

```
#make list of all groups care about
group_df <- ddply(
  deltadat_all, ~category*`sample type`*samplespp*Year, summarise,
  n = length(ednacode))
```

Functions

We define a set of helper functions for subsetting, calculating frequency, and plotting.

```
# basic subsetting and xreffing to deltadat_all to keep samplenames consistent
subset_seq <- function(type, year, seqvar) {
    sampleinfodeltatemp <-
```

```
      deltadat_all[which(deltadat_all$samplespp == type &
                           deltadat_all$Year == year),]
    sND18 <-
      seqvar[, which(colnames(seqvar) %in% sampleinfodeltatemp$ednacode)]
    return(sND18)
}

# calculate frequency of occurrence = number of samples ESV for a given taxa was found in, divided by t
calcFoo <- function(type, year, seqvar){
  sampleinfodeltatemp <-
    deltadat_all[which(deltadat_all$samplespp == type &
                     deltadat_all$Year == year),]
  sND18 <- seqvar[, which(colnames(seqvar) %in% sampleinfodeltatemp$ednacode)]
  # sND18 <- rowSums(sND18 != 0)/ncol(sND18)
  sND18 <- rowSums(sND18 != 0)
  return(sND18)
}

# ellipsess in nmds
veganCovEllipse <- function (cov, center = c(0, 0), scale = 1, npoints = 100)
{
  theta <- (0:npoints) * 2 * pi/npoints
  Circle <- cbind(cos(theta), sin(theta))
  t(center + scale * t(Circle %*% chol(cov)))
}
```

Saving Data and Cleanup

Finally, the current R environment is saved for future use and all temporary objects are removed to free up memory.

```
save.image("preppeddata.RData")
graphics.off()
gc()
```

```
##           used  (Mb) gc trigger  (Mb) limit (Mb) max used  (Mb)
## Ncells 2232170 119.3    4432914 236.8         NA  3174138 169.6
## Vcells 9621187  73.5   23596091 180.1      65536 23596045 180.1
```

```
rm(list=ls())
```
