# Supplementary material for: Looking for the sponge loop: analyses of detritus on a Caribbean forereef using stable isotope and eDNA metabarcoding techniques
Source: PeerJ. 2024 Feb 23;12:e16970. doi: 10.7717/peerj.16970 (PMC10896084; doi:10.7717/peerj.16970)
Supplement: Data S1 — Raw stable isotope measurements are available in SIAdata_30sept2023.xlsx, and code for SIA analyses are available in files starting with “SIMMR_*.Rmd”. The raw sequence tables from metabarcoding are available in .csv files starting with “edna_CO1”, “edna_18S”, and “edna_16S”, and the code to analyze sequence data are in .Rmd files starting with “edna_1_analysis”, “edna_2_analysis”, and “edna_3_analysis”. The pdfs in “markdown_outputs/” subdirectory are retained for quick reference and reproducible by knitting the .Rmds. [file peerj-12-16970-s012.zip › data_peerj/markdown_outputs/edna_3_analysis_detritus_30sept.pdf]

analysis_detritus

Lauren Olinger

9/30/2023

Suction/Tray Detritus Analysis

This section focuses on analyzing the suction/tray detritus data.

Library Imports

Load the necessary R packages.

```
library(readr)
library(plyr)
library(vegan)
```

```
## Loading required package: permute
## Loading required package: lattice
## This is vegan 2.6-2
```

```
library(ggplot2)
library(pairwiseAdonis)
```

```
## Loading required package: cluster
```

```
library(dplyr)
```

```
##
## Attaching package: 'dplyr'
## The following objects are masked from 'package:plyr':
##
##     arrange, count, desc, failwith, id, mutate, rename, summarise,
##     summarize
## The following objects are masked from 'package:stats':
##
##     filter, lag
## The following objects are masked from 'package:base':
##
##     intersect, setdiff, setequal, union
```

```
library(stringr)
```

Load Dataset

```
load("preppeddata.RData")
```

Data Preparation

Subset the data based on the required conditions.

```
groupname <- "detritus"
writeoutfile <- paste("output/", groupname, "/", sep = "")

seqdat <- cbind(
  kingdom = seqdat_kpco$kingdom,
  phylum = seqdat_kpco$phylum,
  class = seqdat_kpco$class,
  order = seqdat_kpco$order,
  subset_seq("suction", "2018", seqdat_kpco),
  subset_seq("suction", "2019", seqdat_kpco),
  subset_seq("tray", "2019", seqdat_kpco)
)
```

Further data manipulation includes creating indices, setting row names, and making a presence-absence matrix.

```
seqind <- str_pad(c(1:nrow(seqdat)), 3, pad = "0")

#make the variable name - store as rownames
rownames(seqdat) <-
  paste(seqind,
        seqdat$kingdom,
        seqdat$phylum,
        seqdat$class,
        seqdat$order,
        sep = "_")

#new trying for sed trap- removing rows with zero identifications
# seqdat <- seqdat[-which(rowSums(seqdat[,5:ncol(seqdat)])==0),]

seqdat$kingdom <- NULL
seqdat$phylum <- NULL
seqdat$class <- NULL
seqdat$order <- NULL

#convert to presence-absence
seqdat[which(seqdat > 0, arr.ind = T)] <- 1

#transpose
seqdat <- t(seqdat)

#get groups from the rownames of seqdat (note this depends on using the same naming schema in ddply, so
# seqdat_groups <-
#   as.factor(sapply(strsplit(rownames(seqdat), "-"), "[[", 1))
seqdat_groups <-
  as.factor(c(
    rep("suction 2018", 5),
    rep("suction 2019", 6),
    rep("tray 2019", 2)
  ))
```

Non-metric Multidimensional Scaling (NMDS)

Perform NMDS to visualize community dissimilarity.

```
# because this is presence/absence, using JACCARD dissimilarity index
# https://www.researchgate.net/post/Does-it-make-any-sense-to-use-both-Jaccard-index-and-Bray-Curtis-co
distmethod <- "jaccard"

seqdat_nmds <- metaMDS(seqdat,
                       distance = distmethod,
                       k = 2)
```

```
## Run 0 stress 0.04627443
## Run 1 stress 0.04861656
## Run 2 stress 0.04338574
## ... New best solution
## ... Procrustes: rmse 0.04490679  max resid 0.09810007
## Run 3 stress 0.04305694
## ... New best solution
## ... Procrustes: rmse 0.07997977  max resid 0.206966
## Run 4 stress 0.04861664
## Run 5 stress 0.04338592
## ... Procrustes: rmse 0.07993111  max resid 0.2099233
## Run 6 stress 0.05961605
## Run 7 stress 0.3160356
## Run 8 stress 0.05815067
## Run 9 stress 0.04305703
## ... Procrustes: rmse 0.0004517258  max resid 0.0008934392
## ... Similar to previous best
## Run 10 stress 0.04627449
## Run 11 stress 0.04338576
## ... Procrustes: rmse 0.08018034  max resid 0.2101808
## Run 12 stress 0.04861663
## Run 13 stress 0.04305704
## ... Procrustes: rmse 0.00046725  max resid 0.0009033268
## ... Similar to previous best
## Run 14 stress 0.04338569
## ... Procrustes: rmse 0.07999963  max resid 0.2099891
## Run 15 stress 0.04861697
## Run 16 stress 0.04305697
## ... Procrustes: rmse 0.0004181295  max resid 0.0008065065
## ... Similar to previous best
## Run 17 stress 0.04627443
## Run 18 stress 0.04305687
## ... New best solution
## ... Procrustes: rmse 0.0002904021  max resid 0.0005539093
## ... Similar to previous best
## Run 19 stress 0.04338576
## ... Procrustes: rmse 0.08009661  max resid 0.2100859
## Run 20 stress 0.04338569
## ... Procrustes: rmse 0.08027872  max resid 0.210274
## *** Solution reached
```

```
seqdat_nmds
```

```
##
```

```
## Call:
## metaMDS(comm = seqdat, distance = distmethod, k = 2)
##
## global Multidimensional Scaling using monoMDS
##
## Data:     seqdat
## Distance: jaccard
##
## Dimensions: 2
## Stress:     0.04305687
## Stress type 1, weak ties
## Two convergent solutions found after 20 tries
## Scaling: centring, PC rotation, halfchange scaling
## Species: expanded scores based on 'seqdat'
```

Several visualizations, including a stress plot and NMDS plot, are generated.

stressplot

```r
stressplot(seqdat_nmds)
```

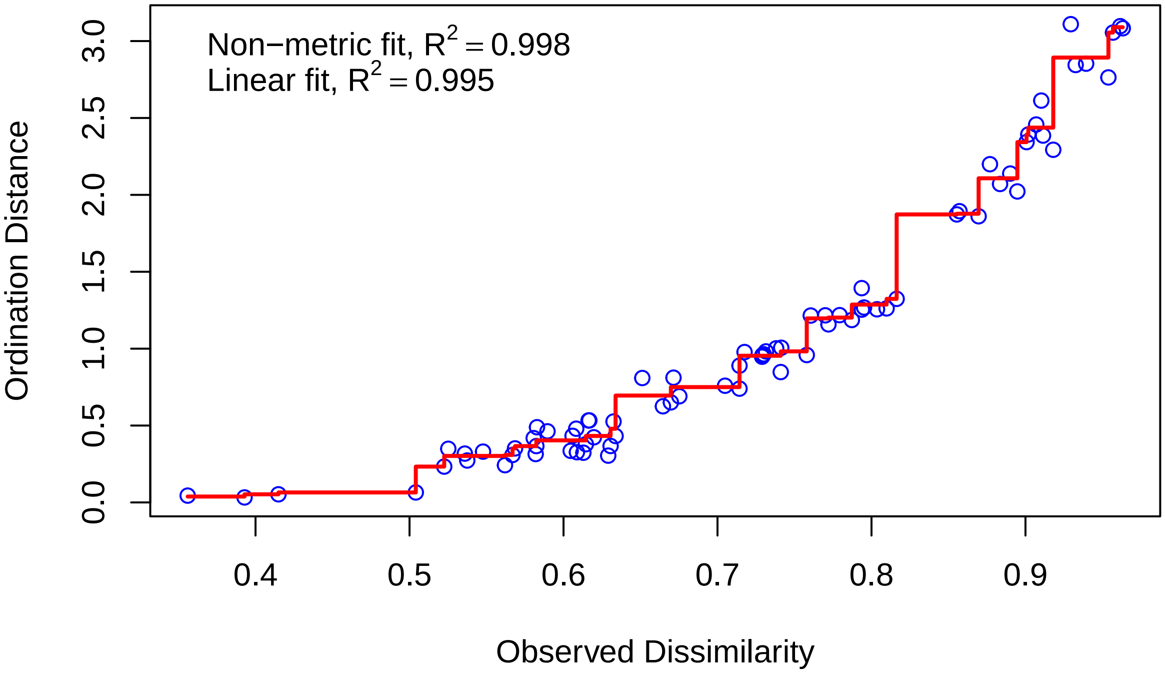

biplot with 95% ellipses

```r
##NMDS points
seqdat_nmds_data <- data.frame(Treatment = seqdat_groups)
seqdat_nmds_data$NMDS1 <- seqdat_nmds$points[, 1]
seqdat_nmds_data$NMDS2 <- seqdat_nmds$points[, 2]

#total abundances for each species
stems <- colSums(seqdat)

#dataframe of species scoes for plotting
spps <- data.frame(scores(seqdat_nmds, display = "species"))

# making a column with species names
```

```
spps$species <- row.names(spps)

#adding the colSums from above
spps$colsums <- stems

#removes NAs
spps <- spps[!is.na(spps$NMDS1) & !is.na(spps$NMDS2), ]

#create an object that is the median of the abundance of the measured species
spps.colmedian <- median(spps$colsums)

#creates a mean instead if you wish to use
spps.colmean <- mean(spps$colsums)

#select the most abundant species. Could discard fewer by going something like - spps$colsums>(spps.col
spps2 <- subset(spps, spps$colsums > spps.colmean)

#otherwise factor doesn't drop unused levels and will throw error
spps2$species <- factor(spps2$species)

# ellipsess in nmds
veganCovEllipse <- function (cov, center = c(0, 0), scale = 1, npoints = 100)
{
  theta <- (0:npoints) * 2 * pi/npoints
  Circle <- cbind(cos(theta), sin(theta))
  t(center + scale * t(Circle %*% chol(cov)))
}


#data for ellipse, in this case using the Treatment factor
ellipse_dat <-
  data.frame() #sets up a data frame before running the function.

for (g in levels(seqdat_nmds_data$Treatment)[1:2]) {
  ellipse_dat <-
    rbind(ellipse_dat, cbind(as.data.frame(
      with(seqdat_nmds_data[seqdat_nmds_data$Treatment == g, ], veganCovEllipse(
        cov.wt(cbind(NMDS1, NMDS2), wt = rep(1 / length(NMDS1), length(NMDS1)))$cov, center =
          c(mean(NMDS1), mean(NMDS2))
      ))
    ), Treatment = g))
}


#make sure levels match up (they should)
levels(seqdat_nmds_data$Treatment)
```

```
## [1] "suction 2018" "suction 2019" "tray 2019"
```

```
levels(ellipse_dat$Treatment)
```

```
## NULL
```

```
ellipse_dat$Treatment <-
  factor(ellipse_dat$Treatment, levels = levels(seqdat_nmds_data$Treatment))
```

```r
#plotting.

#ellipse
g1 <-
  ggplot(data = seqdat_nmds_data, aes(y = NMDS2, x = NMDS1)) +
  geom_polygon(
    data = ellipse_dat,
    aes(
      x = NMDS1,
      y = NMDS2,
      group = Treatment,
      color = Treatment
    ),
    alpha = 0,
    size = 1,
    show.legend = F
  )
```

```
## Warning: Using `size` aesthetic for lines was deprecated in ggplot2 3.4.0.
## i Please use `linewidth` instead.
## This warning is displayed once every 8 hours.
## Call `lifecycle::last_lifecycle_warnings()` to see where this warning was
## generated.
```

```r
#points
g5 <-
  g1 +
  geom_point(aes(colour = Treatment), alpha = 1, size = 2) +
  scale_colour_manual(values = c("firebrick1", "firebrick3", "black"))

#cleaning it up, makin everything big and readible
nmds_ggplot <- g5 +
  theme_light() +
  theme(plot.title = element_text(size = 12, face = "bold"))  +
  theme(legend.title = element_text(
    size = 12,
    face = "bold"
    )) +
  theme(
    legend.position = "bottom",
    legend.direction = "horizontal",
    legend.box = "horizontal"
  ) +
  theme(legend.text = element_text(size = 12)) +
  theme(axis.text = element_text(size = 12)) +
  theme(axis.title = element_text(
    size = 12,
    face = "bold"
  )) +
  theme(legend.title = element_blank())

nmds_ggplot
```

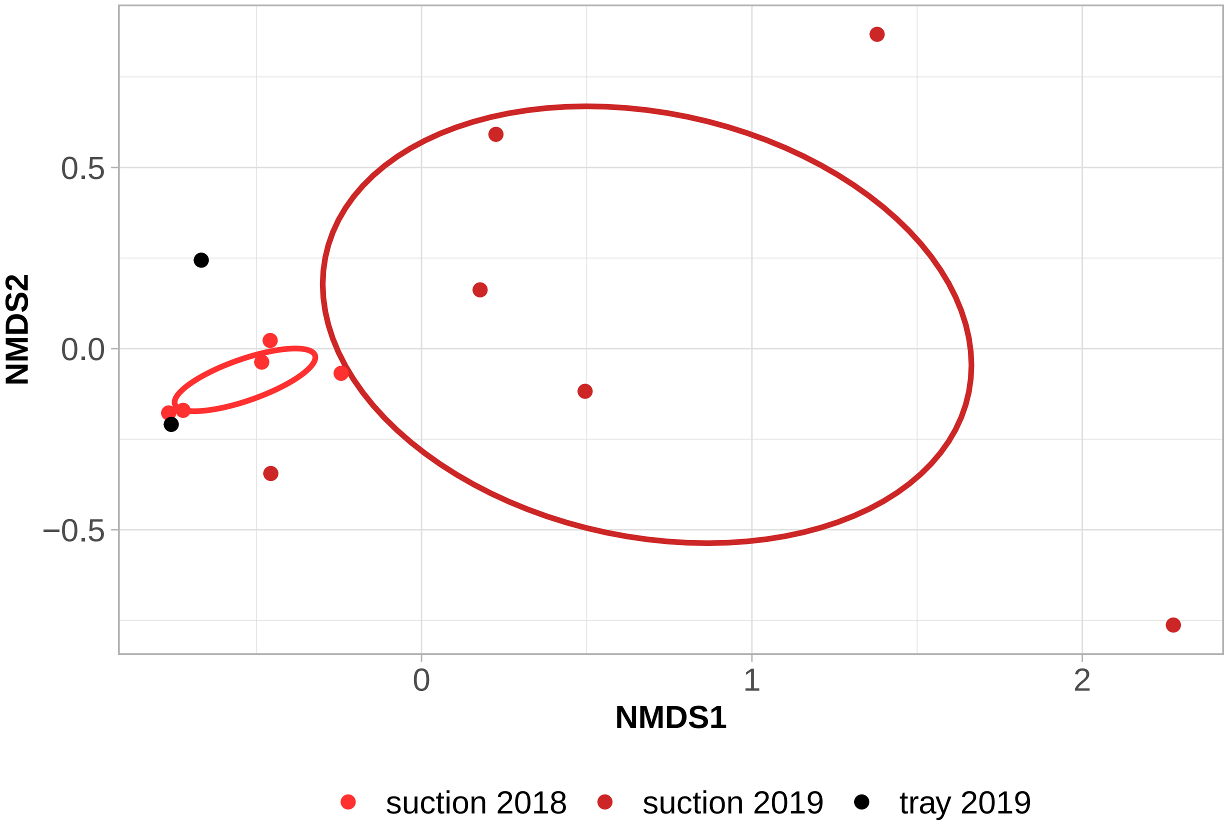


```r
#remove stuff
rm(
  g1,
  g5,
  spps,
  spps2,
  ellipse_dat,
  seqdat_nmds_data,
  spps.colmean,
  spps.colmedian,
  stems
)
```

PERMANOVA Analysis

Statistical testing for community dissimilarity across groups.

```r
dissim_jaccard <- vegdist(seqdat, method = distmethod)
perm <- adonis2(dissim_jaccard ~ seqdat_groups, data = data.frame(seqdat))

densityplot(permustats(perm))
```

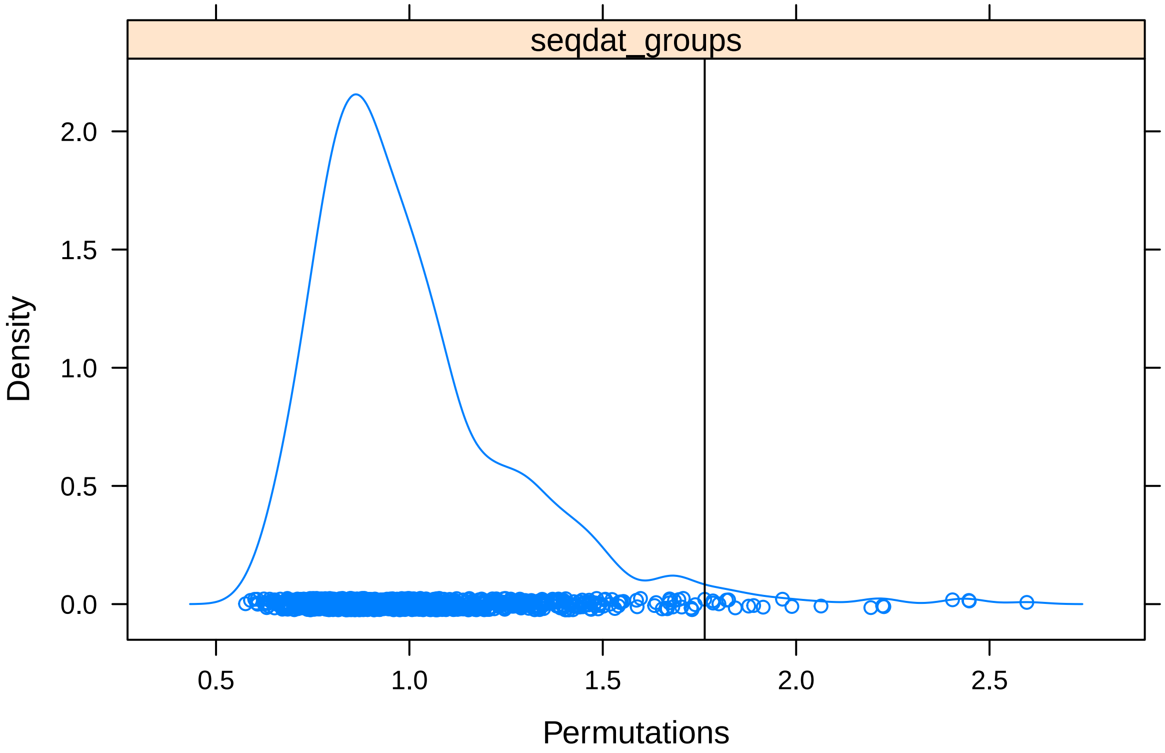

Pairwise comparisons using PERMANOVA.

```
#using pairwise adonis behind : https://github.com/pmartinezarbizu/pairwiseAdonis
# Sys.setenv(R_REMOTES_NO_ERRORS_FROM_WARNINGS=TRUE)
# library(devtools)
# install_github("pmartinezarbizu/pairwiseAdonis/pairwiseAdonis")

perm_pairwise <-
  pairwiseAdonis::pairwise.adonis(dissim_jaccard, seqdat_groups)
```

```
## Set of permutations < 'minperm'. Generating entire set.
```

```
perm_pairwise[, 3:5] <- round(perm_pairwise[, 3:5], 2)
perm_pairwise
```

```
##                          pairs Df SumsOfSqs F.Model   R2 p.value p.adjusted sig
## 1 suction 2018 vs suction 2019  1      0.57    2.32 0.21   0.011      0.033   .
## 2    suction 2018 vs tray 2019  1      0.18    1.14 0.19   0.478      1.000
## 3    suction 2019 vs tray 2019  1      0.42    1.44 0.19   0.161      0.483
```

Similarity Percentages (SIMPER) Analysis

Determine which species contribute most to the dissimilarity between groups.

```
simper_seq <- simper(seqdat, permutations = 999, group = seqdat_groups)
```

```
simperSummary <- summary(simper_seq)

simperSummary <- simperSummary$`suction 2018_suction 2019`

simperSummary <- simperSummary[order(rownames(simperSummary)), ]
```

```
simperSummary <- cbind(
  kingdom = seqdat_kpco$kingdom,
  phylum = seqdat_kpco$phylum,
  class = seqdat_kpco$class,
  order = seqdat_kpco$order,
  simperSummary
)

sum(simperSummary$average)
```

```
## [1] 0.6324202
```

Cleanup

Clear graphics and memory.

```
graphics.off()
gc()
```

```
##            used  (Mb) gc trigger  (Mb) limit (Mb) max used  (Mb)
## Ncells  2411106 128.8    4239935 226.5         NA  3968825 212.0
## Vcells 10195982  77.8   17518488 133.7      65536 14531905 110.9
```

```
rm(list = ls())
```
