# Supplementary material for: Looking for the sponge loop: analyses of detritus on a Caribbean forereef using stable isotope and eDNA metabarcoding techniques
Source: PeerJ. 2024 Feb 23;12:e16970. doi: 10.7717/peerj.16970 (PMC10896084; doi:10.7717/peerj.16970)
Supplement: Data S1 — Raw stable isotope measurements are available in SIAdata_30sept2023.xlsx, and code for SIA analyses are available in files starting with “SIMMR_*.Rmd”. The raw sequence tables from metabarcoding are available in .csv files starting with “edna_CO1”, “edna_18S”, and “edna_16S”, and the code to analyze sequence data are in .Rmd files starting with “edna_1_analysis”, “edna_2_analysis”, and “edna_3_analysis”. The pdfs in “markdown_outputs/” subdirectory are retained for quick reference and reproducible by knitting the .Rmds. [file peerj-12-16970-s012.zip › data_peerj/markdown_outputs/edna_2_analysis_summarytable_30sept.pdf]

# analysis_summarytable

Lauren Olinger

9/30/2023

## Introduction

This analysis is designed for subsetting, analyzing, and summarizing genetics data related to `suction/tray detritus`.

## Library Loading

```r
library(readr)
library(plyr)
library(vegan)
```

```
## Loading required package: permute

## Loading required package: lattice

## This is vegan 2.6-2
```

```r
library(ggplot2)
library(pairwiseAdonis)
```

```
## Loading required package: cluster
```

```r
library(dplyr)
```

```
##
## Attaching package: 'dplyr'

## The following objects are masked from 'package:plyr':
##
##     arrange, count, desc, failwith, id, mutate, rename, summarise,
##     summarize

## The following objects are masked from 'package:stats':
##
##     filter, lag

## The following objects are masked from 'package:base':
##
##     intersect, setdiff, setequal, union
```

```r
library(reshape)
```

```
##
## Attaching package: 'reshape'

## The following object is masked from 'package:dplyr':
##
##     rename
```

```
## The following objects are masked from 'package:plyr':
##
##     rename, round_any
```

## Data Loading

Load the previously saved R data file `preppeddata.RData`

```
load("preppeddata.RData")
```

## Function Definition for Tabulating ESVs

Define the `tabESVs` function to tabulate the number of Exact Sequence Variants (ESVs) for each sample type at various hierarchical organizations.

```
# tabulate number of exact sequence variants (ESVs) for each sample type at each hierarchical organizat
tabESVs <- function(seqvar){
  newdf = data.frame(firstcol = matrix(ncol = 1, nrow = nrow(seqvar)))

  for (i in 1:length(unique(group_df$samplespp))){
    for (j in 1:length(unique(group_df$Year))){
      spi <- unique(group_df$samplespp)[i]
      yrj <- unique(group_df$Year)[j]
      name <- paste(spi,yrj,sep="-")
      if (any(group_df$samplespp == spi & group_df$Year == yrj)==FALSE){
        next
      }
      tempdf <- data.frame(subset_seq(spi, yrj, seqvar))
      if (ncol(tempdf)>1){
        newvar <- rowSums(subset_seq(spi, yrj, seqvar))
      } else {
        newvar <- tempdf
      }
      newdf <- cbind(newdf, newvar)
      colnames(newdf)[ncol(newdf)] <- name
    }
  }
  newdf$firstcol <- NULL
  return(newdf)
}
```

## Summarization of Data

Use the `tabESVs` function to summarize the data at different levels of organization.

```
seqdatsum <- cbind(
  seqdat_info,
  tabESVs(seqdat_all)
)

seqdatsumkp <-
  cbind(kingdom = seqdat_kp$kingdom,
        phylum = seqdat_kp$phylum,
        tabESVs(seqdat_kp))
```

```
seqdatsumkpco <-
  cbind(kingdom = seqdat_kpco$kingdom,
        phylum = seqdat_kpco$phylum,
        class = seqdat_kpco$class,
        order = seqdat_kpco$order,
        tabESVs(seqdat_kpco))

seqdatsumkpcofgs <-
  cbind(kingdom = seqdat_kpcofgs$kingdom,
        phylum = seqdat_kpcofgs$phylum,
        class = seqdat_kpcofgs$class,
        order = seqdat_kpcofgs$order,
        family = seqdat_kpcofgs$family,
        genus = seqdat_kpcofgs$genus,
        species = seqdat_kpcofgs$species,
        tabESVs(seqdat_kpcofgs))
```

## Function Definition for Tabulating FOOs

Define the `tabFOOs` function to compute the Frequency Of Occurrence (FOO) for each sample type at each hierarchical organization.

```
# tabulate frequency of occurrence (FOO) for each sample type at each hierarchical organization
tabFOOs <- function(seqvar){
  newdf = data.frame(firstcol = matrix(ncol = 1, nrow = nrow(seqvar)))

  for (i in 1:length(unique(group_df$samplespp))){
    for (j in 1:length(unique(group_df$Year))){
      spi <- unique(group_df$samplespp)[i]
      yrj <- unique(group_df$Year)[j]
      name <- paste(spi,yrj,sep="-")
      if (any(group_df$samplespp == spi & group_df$Year == yrj)==FALSE){
        next
      }
      tempdf <- data.frame(subset_seq(spi, yrj, seqvar))
      if (ncol(tempdf)>1){
        newvar <- calcFoo(spi, yrj, seqvar)
      } else {
        tempdf[which(tempdf>0),] <- 1
        newvar <- tempdf
      }
      newdf <- cbind(newdf, newvar)
      colnames(newdf)[ncol(newdf)] <- name
    }
  }
  newdf$firstcol <- NULL
  return(newdf)
}
```

## Summarization of FOO Data

Use the `tabFOOs` function to summarize the data at different levels of organization.

```
seqdatfoo <- cbind(
  seqdat_info,
  tabFOOs(seqdat_all)
)

seqdatfookp <-
  cbind(kingdom = seqdat_kp$kingdom,
        phylum = seqdat_kp$phylum,
        tabFOOs(seqdat_kp))

seqdatfookpco <-
  cbind(kingdom = seqdat_kpco$kingdom,
        phylum = seqdat_kpco$phylum,
        class = seqdat_kpco$class,
        order = seqdat_kpco$order,
        tabFOOs(seqdat_kpco))

seqdatfookpcofgs <-
  cbind(kingdom = seqdat_kpcofgs$kingdom,
        phylum = seqdat_kpcofgs$phylum,
        class = seqdat_kpcofgs$class,
        order = seqdat_kpcofgs$order,
        family = seqdat_kpcofgs$family,
        genus = seqdat_kpcofgs$genus,
        species = seqdat_kpcofgs$species,
        tabFOOs(seqdat_kpcofgs))
```

## Data Export

Export several summary tables to the `output/summarytables/` directory.

```
write.csv(group_df, "output/summarytables/groupdf.csv")

write.csv(seqdatsum,"output/summarytables/seqdatsum.csv")
write.csv(seqdatsumkp,"output/summarytables/seqdatsumkp.csv")
write.csv(seqdatsumkpco,"output/summarytables/seqdatsumkpco.csv")
write.csv(seqdatsumkpcofgs,"output/summarytables/seqdatsumkpcofgs.csv")

write.csv(seqdatfoo,"output/summarytables/seqdatfoo.csv")
write.csv(seqdatfookp,"output/summarytables/seqdatfookp.csv")
write.csv(seqdatfookpco,"output/summarytables/seqdatfookpco.csv")
write.csv(seqdatfookpcofgs,"output/summarytables/seqdatfookpcofgs.csv")
```

## Data Reshaping: seqdatfookp

This section focuses on reshaping the dataset `seqdatfookp`.

### Creating Additional Rows for `seqdatfookp`

First, we generate some additional rows that represent a summarized view (`totaln`) and then bind these rows to the original data.

```
addtoseqdatfookp <- cbind(
  kingdom = "totaln",
```

```
  phylum = "totaln",
  t(group_df$n)
)

colnames(addtoseqdatfookp) <- colnames(seqdatfookp)

seqdatfookp <- rbind(seqdatfookp, addtoseqdatfookp)
```

### Melting `seqdatfookp`

Next, we reshape the data from a wide format to a long format for easier visualization and analysis.

```
meltseqdatfookp <- melt(
  seqdatfookp,
  measure.vars = c(3:ncol(seqdatfookp)),
  variable.name = "sampletype",
  value.name = "nSamples",
  na.rm = TRUE
)
```

### Parsing Variable Names

Subsequently, we dissect the variable names from the melted data to derive sample types, species, and year.

```
newvar <- as.character(meltseqdatfookp$variable)

newvarsampletypespp <- as.character(as.factor(sapply(strsplit(newvar, "-"), "[[", 1)))
newvaryear <- as.character(sapply(strsplit(newvar, "-"), "[[", 2))
newvarsampletype <- as.character(sapply(strsplit(newvarsampletypespp, "\\("), "[[", 1))

newvarspp <- rep(NA, length(newvarsampletypespp))
newvarspp[grep("\\(", newvarsampletypespp)] <-
  as.character(as.factor(sapply(
    strsplit(newvarsampletypespp[grep("\\(", newvarsampletypespp)], "\\("), "[[", 2
  )))
newvarspp <- gsub("\\)","",newvarspp)
```

### Finalizing the Reshaped Data

Lastly, we bind the parsed variables to the melted dataset and export it.

```
meltseqdatfookp <- cbind(
  kingdom = meltseqdatfookp$kingdom,
  phylum = meltseqdatfookp$phylum,
  sampletype = newvarsampletype,
  spp = newvarspp,
  year = newvaryear,
  value = meltseqdatfookp$value
)

write.csv(meltseqdatfookp,"output/summarytables/meltseqdatfookp.csv")
```

## Data Reshaping: seqdatfookpcofgs

This section focuses on reshaping the dataset `seqdatfookpcofgs`.

### Creating Additional Rows for `seqdatfookpcofgs`

Similar to before, we generate some additional rows for a summarized view (`totaln`).

```
addtoseqdatfookpcofgs <- cbind(
  kingdom = "totaln",
  phylum = "totaln",
  class = "totaln",
  order = "totaln",
  family = "totaln",
  genus = "totaln",
  species = "totaln",
  t(group_df$n)
)

colnames(addtoseqdatfookpcofgs) <- colnames(seqdatfookpcofgs)
seqdatfookpcofgs <- rbind(seqdatfookpcofgs, addtoseqdatfookpcofgs)
```

### Melting `seqdatfookpcofgs`

Now, we melt this dataset to transform it from a wide to a long format.

```
meltseqdatfookpcofgs <- melt(
  seqdatfookpcofgs,
  measure.vars = c(8:ncol(seqdatfookpcofgs)),
  variable.name = "sampletype",
  value.name = "nSamples",
  na.rm = TRUE
)
```

### Parsing Variable Names

Once again, we parse out details from variable names for deeper insights.

```
newvar <- as.character(meltseqdatfookpcofgs$variable)

newvarsampletypespp <- as.character(as.factor(sapply(strsplit(newvar, "-"), "[[", 1)))
newvaryear <- as.character(sapply(strsplit(newvar, "-"), "[[", 2))
newvarsampletype <- as.character(sapply(strsplit(newvarsampletypespp, "\\("), "[[", 1))

newvarspp <- rep(NA, length(newvarsampletypespp))
newvarspp[grep("\\(", newvarsampletypespp)] <-
  as.character(as.factor(sapply(
    strsplit(newvarsampletypespp[grep("\\(", newvarsampletypespp)], "\\("), "[[", 2
  )))
newvarspp <- gsub("\\)","",newvarspp)
```

### Finalizing the Reshaped Data

Finally, combine the parsed variables with the melted dataset and export.

```
meltseqdatfookpcofgs <- cbind(
  kingdom = meltseqdatfookpcofgs$kingdom,
  phylum = meltseqdatfookpcofgs$phylum,
  class = meltseqdatfookpcofgs$class,
  order = meltseqdatfookpcofgs$order,
  family = meltseqdatfookpcofgs$family,
```

```
  genus = meltseqdatfookpcofgs$genus,
  species = meltseqdatfookpcofgs$species,
  sampletype = newvarsampletype,
  spp = newvarspp,
  year = newvaryear,
  value = meltseqdatfookpcofgs$value
)

write.csv(meltseqdatfookpcofgs,"output/summarytables/meltseqdatfookpcofgs.csv")
```

## Cleanup

Remove all the variables created during this script.

```
rm(list=ls())
```
