# Supplementary material for: Looking for the sponge loop: analyses of detritus on a Caribbean forereef using stable isotope and eDNA metabarcoding techniques
Source: PeerJ. 2024 Feb 23;12:e16970. doi: 10.7717/peerj.16970 (PMC10896084; doi:10.7717/peerj.16970)
Supplement: Data S1 — Raw stable isotope measurements are available in SIAdata_30sept2023.xlsx, and code for SIA analyses are available in files starting with “SIMMR_*.Rmd”. The raw sequence tables from metabarcoding are available in .csv files starting with “edna_CO1”, “edna_18S”, and “edna_16S”, and the code to analyze sequence data are in .Rmd files starting with “edna_1_analysis”, “edna_2_analysis”, and “edna_3_analysis”. The pdfs in “markdown_outputs/” subdirectory are retained for quick reference and reproducible by knitting the .Rmds. [file peerj-12-16970-s012.zip › data_peerj/markdown_outputs/SIMMR_2019_Tray_Combine_alg_Combine_herb.pdf]

SIMMR: 2019 Tray (pooled alg & herbivore)

Lauren Olinger

9/30/2023

Load libraries for SIMMR & import data

```
library(simmr)
library(plyr)
```

import data and define variable name

```
#to combine variables
database <- read.csv('allData_28june.csv') # identical to sheet in SIAdata_30sept2023.xlsx

#omit samples with omit == y if d13C > -10 or if not enough material on filter
database <- database[which(database$omit=="n"),]

#define prefix of output files
filePrefix <- "figs/2019_Tray_Combine_alg_Combine_herb_"
```

extract mix and source data

```
#make mix - the mixture of detritus end member samples - as individual samplings
mix <- as.matrix(database[which(database$category=="detritus" &
                                  database$Year=="2019" &
                                database$Source == "tray detritus"),7:8])

#make sources - the various contributing sources , as mean and SD values-
# for 2018 - combine alg tissue and herbivore feces
sources <- database[which(database$Year==2019 & database$category=="end member"),]

# #combine alg tissue
sources$Source[which(sources$Source=="algae tissue (Dict)"|
                          sources$Source=="algae tissue (Hali)"|
                          sources$Source=="algae tissue (Lobo)")] <- "algae tissue"

#combine herbivore feces
sources$Source[which(sources$Source=="herbivore feces (Abah)"|
                          sources$Source=="herbivore feces (Acoe)")] <- "herbivore feces"

#combine spongivore feces
sources$Source[which(sources$Source=="spongivore feces (Hcil)"|
                          sources$Source=="spongivore feces (Ppar)")] <-
"spongivore feces"
```

```
#summarize sources
sources <-
  ddply(sources, ~Source,
        summarise,
        Meand13C = mean(as.numeric(d13C)),
        SDd13C = sd(as.numeric(d13C)),
        Meand15N = mean(as.numeric(d15N)),
        SDd15N = sd(as.numeric(d15N)),
        color = color[1],
        order = order[1]
        )

sources <- sources[order(sources$order),]
# sources$Source <- sources$sample.type

if(any(is.na(sources))){
  sources[which(is.na(sources),arr.ind = T)] <- 0
}
```

preparing variable mix for the source data

```
s_names <- as.character(sources$Source)
s_means <- cbind(sources$Meand13C, sources$Meand15N)
s_sds <- cbind(sources$SDd13C, sources$SDd15N)

simmr_in = simmr_load(mixtures= mix,
                      source_names=s_names,
                      source_means=s_means,
                      source_sds=s_sds)

simmr_in
```

```
## This is a valid simmr input object with 3 observations, 2 tracers, and 11 sources.
## The source names are: algae tissue, BCM tissue, herbivore feces, spongivore feces, emergent sponge t
## The tracer names are: d13C, d15N.
```

biplot

```
plot(simmr_in,
     xlab= expression(paste(delta^13, "C (\u2030)",sep="")),
     ylab= expression(paste(delta^15, "N (\u2030)",sep="")),
     ggargs = list(xlim(-28,-9), ylim(-3,6)),
     xlim = c(-29,-2),
     ylim = c(-1.5,6),
     title='', mix_name = "EAM detritus") +
  scale_color_manual(values = c("black", sources$color)) +
  theme_classic()
```

```
## Scale for colour is already present.
## Adding another scale for colour, which will replace the existing scale.
```

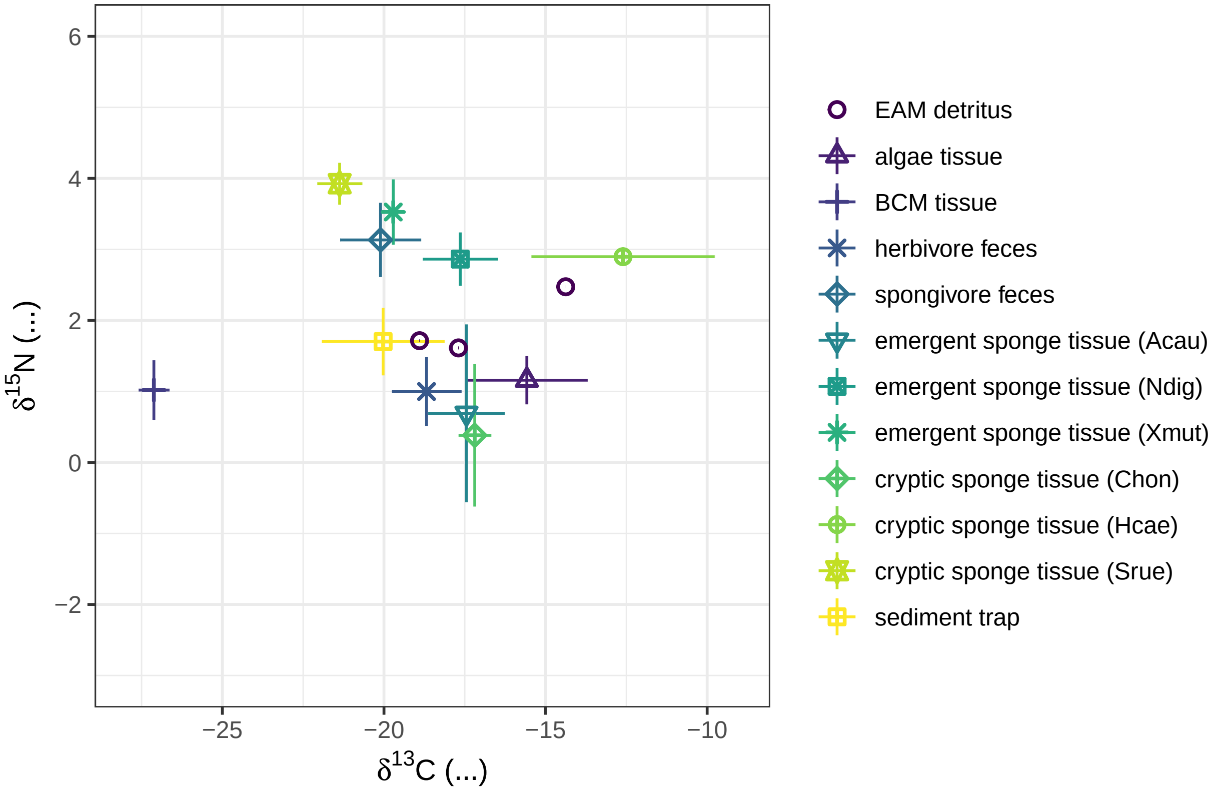

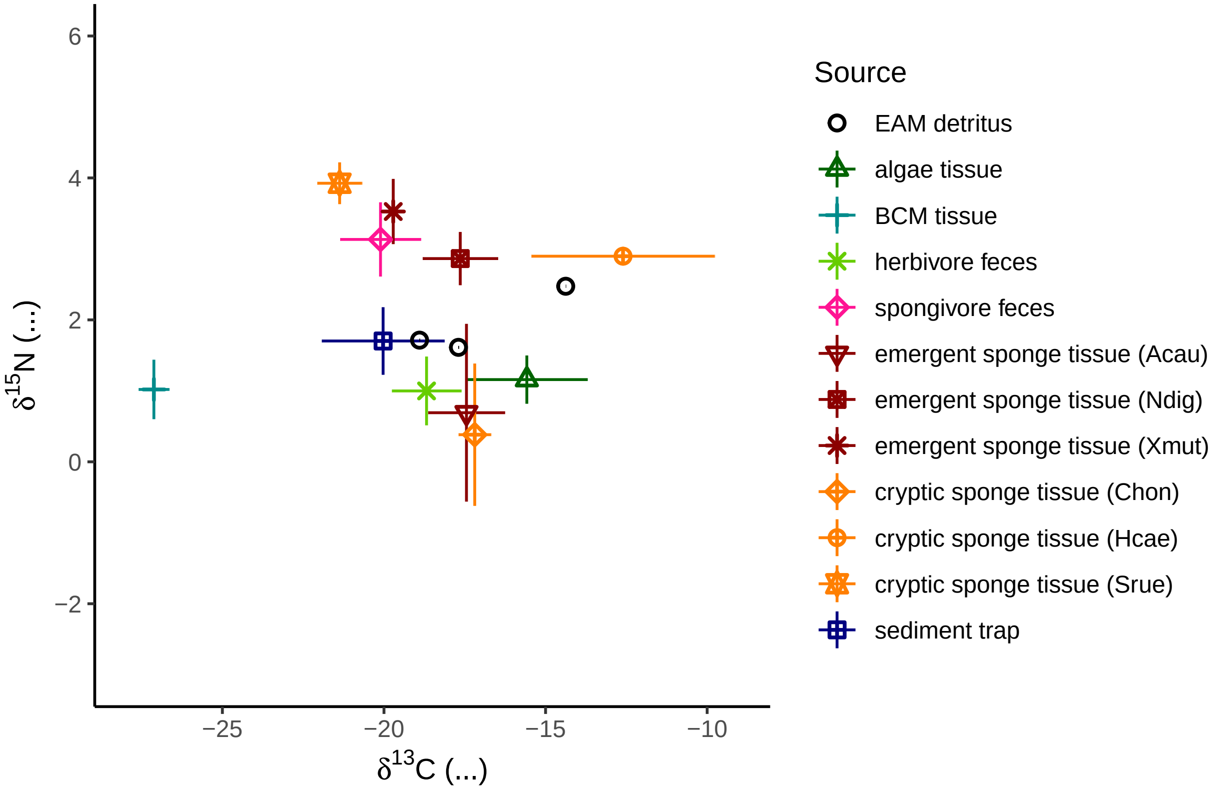

run simmr model

```
simmr_out = simmr_mcmc(simmr_in)
```

```
## module glm loaded

## Compiling model graph
##    Resolving undeclared variables
##    Allocating nodes
## Graph information:
##    Observed stochastic nodes: 6
##    Unobserved stochastic nodes: 13
##    Total graph size: 209
##
## Initializing model
```

simmr diagnostics

If the model run has converged properly the values should be close to 1. If they are above 1.1, we recommend a longer run. See help(simmr_mcmc) for how to do this. The values in this example seem to have converged well.

```
summary(simmr_out,type='diagnostics')
```

```
##
## Summary for 1
## Gelman diagnostics - these values should all be close to 1.
## If not, try a longer run of simmr_mcmc.
##                      deviance                   algae tissue
##                             1                              1
##                    BCM tissue                herbivore feces
##                             1                              1
##             spongivore feces emergent sponge tissue (Acau)
##                             1                              1
## emergent sponge tissue (Ndig) emergent sponge tissue (Xmut)
##                             1                              1
##  cryptic sponge tissue (Chon)  cryptic sponge tissue (Hcae)
##                             1                              1
##  cryptic sponge tissue (Srue)                  sediment trap
##                             1                              1
##                     sd[d13C]                       sd[d15N]
##                             1                              1
```

statistics

simmr produces both textual and graphical summaries of the model run. Starting with the textual summaries, we can get tables of the means, standard deviations and credible intervals (the Bayesian equivalent of a confidence interval) with:

```
summary(simmr_out,type='statistics')
```

cleaned up summary table with the percentages of contribution to the detritus pool

```
summary <- round(summary(simmr_out,type='statistics')$statistics,3)*100
```

```
##
## Summary for 1
##                                 mean    sd
## deviance                      21.297 3.827
## algae tissue                   0.108 0.102
## BCM tissue                     0.071 0.061
```

```
## herbivore feces                 0.092 0.087
## spongivore feces                0.083 0.076
## emergent sponge tissue (Acau)   0.099 0.090
## emergent sponge tissue (Ndig)   0.092 0.087
## emergent sponge tissue (Xmut)   0.078 0.068
## cryptic sponge tissue (Chon)    0.101 0.089
## cryptic sponge tissue (Hcae)    0.109 0.098
## cryptic sponge tissue (Srue)    0.074 0.063
## sediment trap                   0.092 0.091
## sd[d13C]                        5.026 5.263
## sd[d15N]                        0.845 1.060
```

These suggest that the proportions for this model lean towards the "algae/BCM/herbivore feces/sed trap" group.

can produce table of quantiles:

```
summary(simmr_out,type='quantiles')
```

```
##
## Summary for 1
##                                   2.5%    25%    50%    75%  97.5%
## deviance                        16.221 18.563 20.484 23.135 31.113
## algae tissue                     0.009  0.037  0.074  0.144  0.389
## BCM tissue                       0.008  0.029  0.053  0.094  0.240
## herbivore feces                  0.009  0.032  0.064  0.123  0.330
## spongivore feces                 0.008  0.030  0.059  0.109  0.290
## emergent sponge tissue (Acau)    0.009  0.035  0.070  0.136  0.336
## emergent sponge tissue (Ndig)    0.008  0.032  0.065  0.121  0.333
## emergent sponge tissue (Xmut)    0.008  0.031  0.057  0.103  0.268
## cryptic sponge tissue (Chon)     0.009  0.036  0.073  0.141  0.335
## cryptic sponge tissue (Hcae)     0.009  0.037  0.079  0.150  0.374
## cryptic sponge tissue (Srue)     0.008  0.028  0.055  0.099  0.245
## sediment trap                    0.008  0.031  0.061  0.121  0.348
## sd[d13C]                         1.161  2.363  3.489  5.630 19.089
## sd[d15N]                         0.073  0.345  0.569  0.954  3.539
```

density plot

simmr can also produce histograms, boxplots, density plots, and matrix plots of the output. Starting with the density plot:

```
plot(simmr_out,type='density') +
  scale_fill_manual(values=sources$color)
```

```
## Scale for fill is already present.
## Adding another scale for fill, which will replace the existing scale.
```

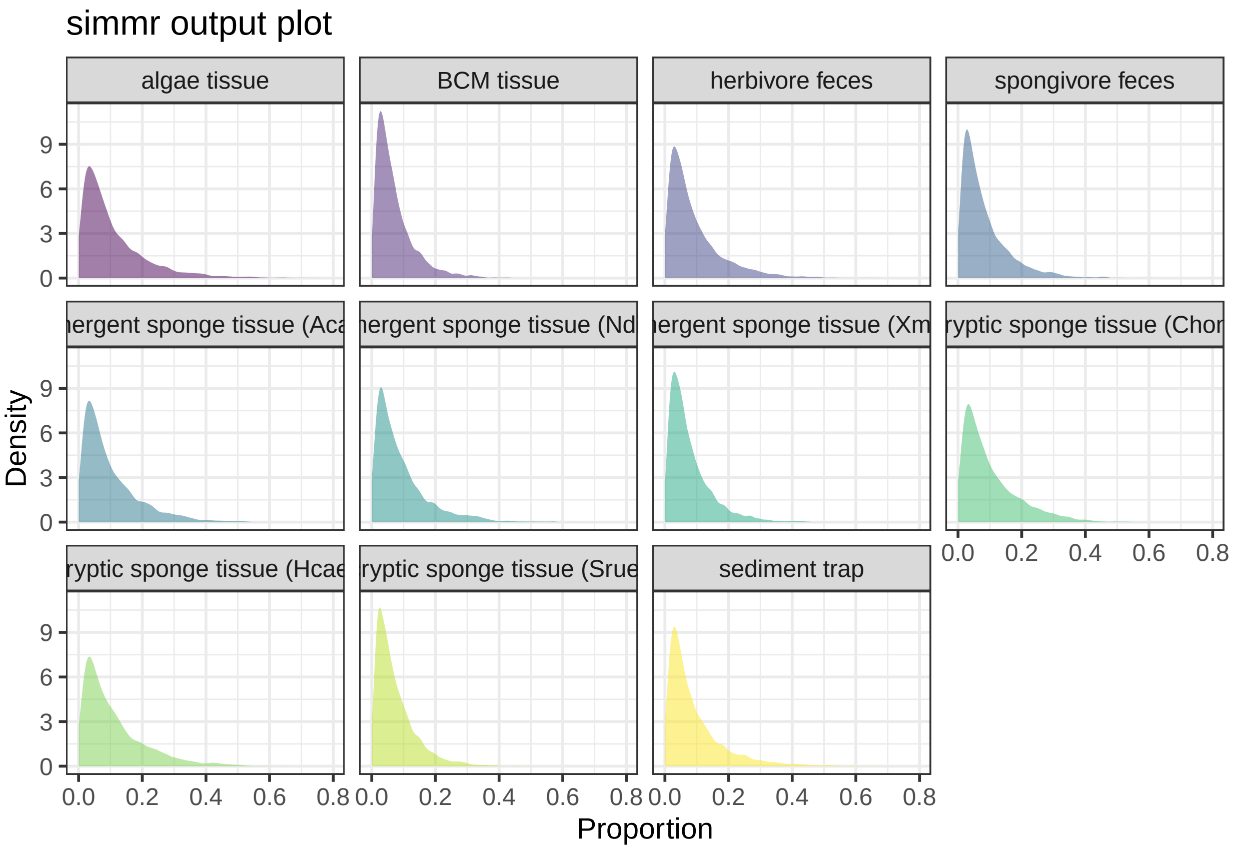

simmr output plot
algae tissue
BCM tissue
herbivore feces
spongivore feces
ıergent sponge tissue (Aca
ıergent sponge tissue (Nd
ıergent sponge tissue (Xm
ryptic sponge tissue (Chor
ryptic sponge tissue (Hcae
ryptic sponge tissue (Srue
sediment trap
Density
Proportion

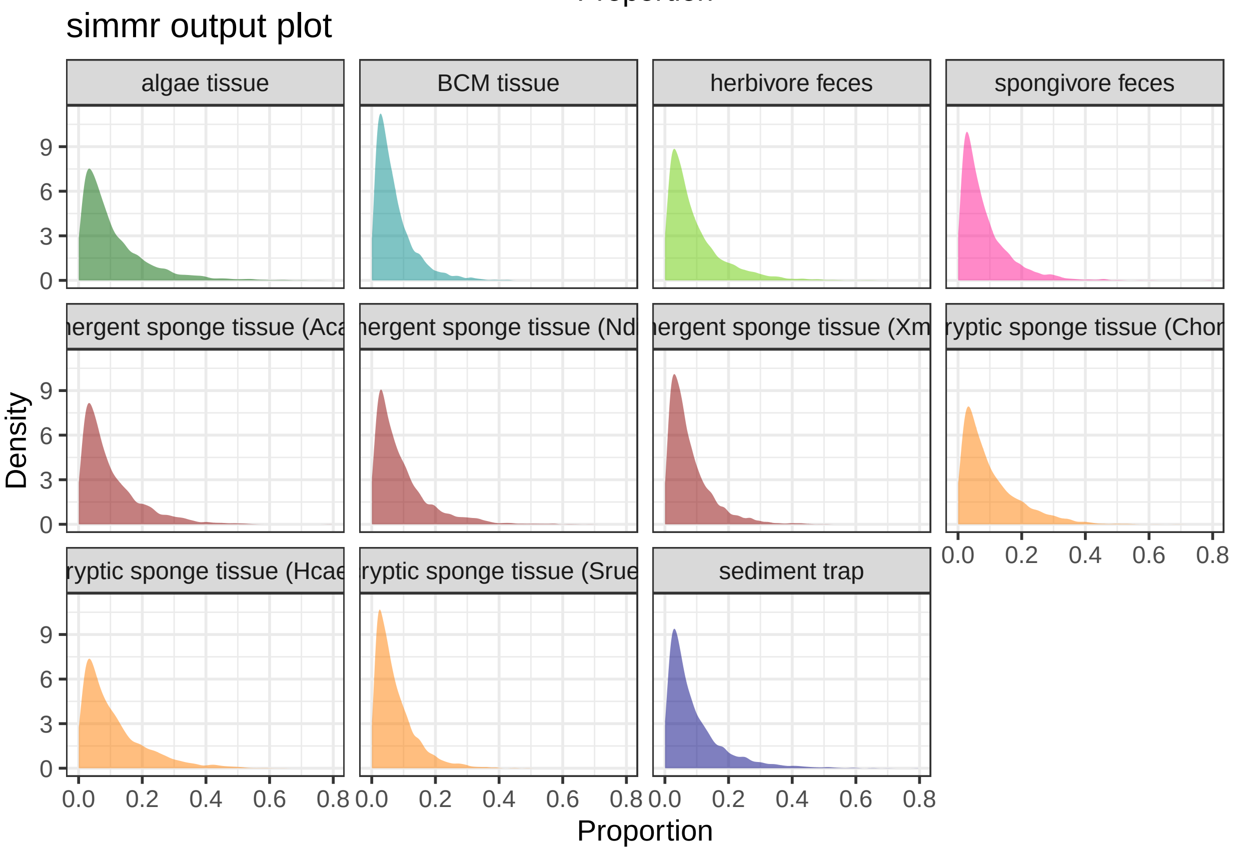

simmr output plot
algae tissue
BCM tissue
herbivore feces
spongivore feces
ıergent sponge tissue (Aca
ıergent sponge tissue (Nd
ıergent sponge tissue (Xm
ryptic sponge tissue (Chor
ryptic sponge tissue (Hcae
ryptic sponge tissue (Srue
sediment trap
Density
Proportion

matrix plot

The most useful output plot is the matrix plot:

This shows the source histograms on the diagonal, contour plots of the relationship between the sources on the upper diagonal, and the correlation between the sources on the lower diagonal.

```r
plot(simmr_out,type='matrix')
```

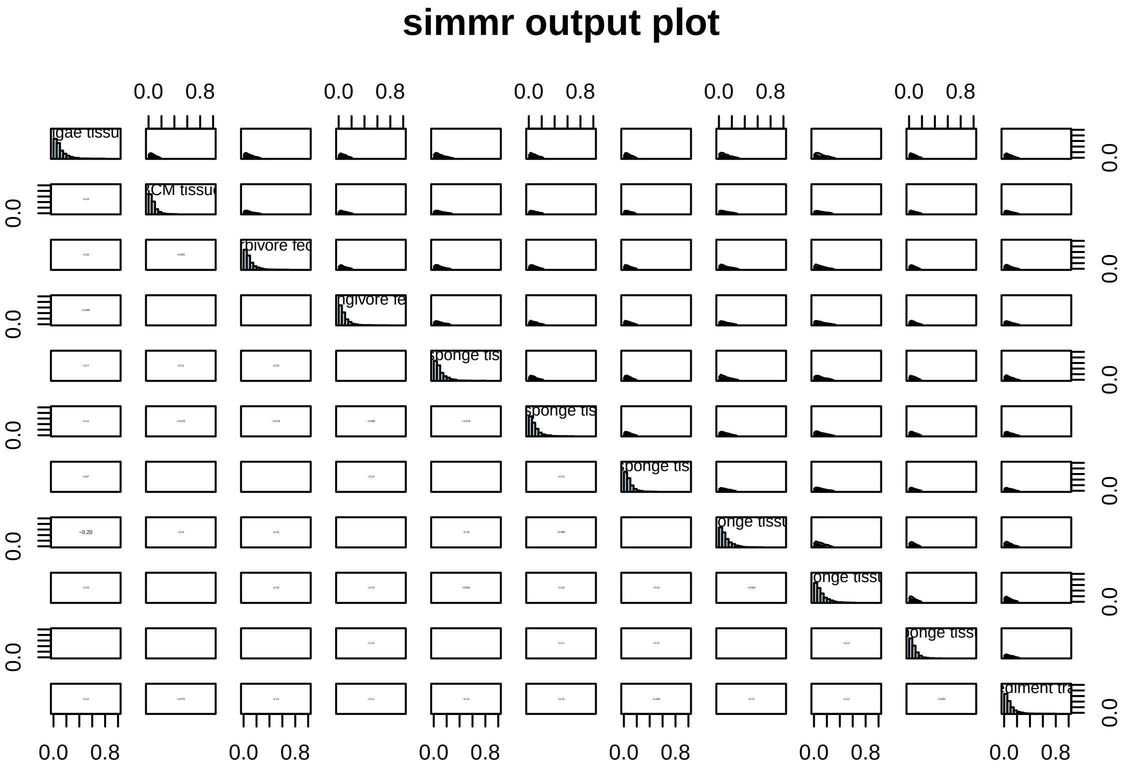

Large negative correlations indicate that the model cannot discern between the two sources; they may lie close together in iso-space. Large positive correlations are also possible when mixture data lie in a polygon consisting of multiple competing sources.

boxplot

boxplot of contribution of each source item to detrital signature

```r
plot(simmr_out, type = "boxplot", title = "", alpha = 1) +
  scale_fill_manual(values = c(sources$color)) +
  scale_x_discrete(limits = rev)+
  theme_classic()+
  theme(legend.position = "none")
```

```
## Scale for fill is already present.
## Adding another scale for fill, which will replace the existing scale.
```

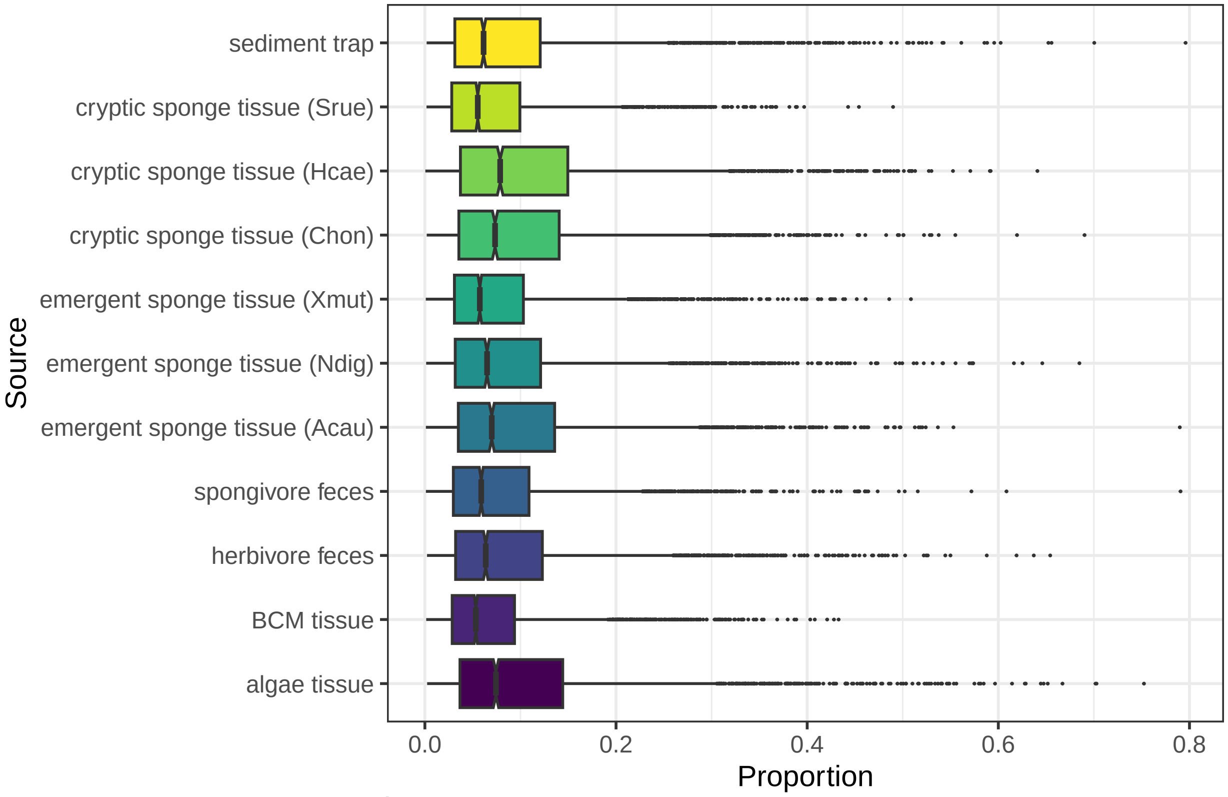
sediment trap
cryptic sponge tissue (Srue)
cryptic sponge tissue (Hcae)
cryptic sponge tissue (Chon)
emergent sponge tissue (Xmut)
emergent sponge tissue (Ndig)
emergent sponge tissue (Acau)
spongivore feces
herbivore feces
BCM tissue
algae tissue
Source
0.0
0.2
0.4
0.6
0.8
Proportion

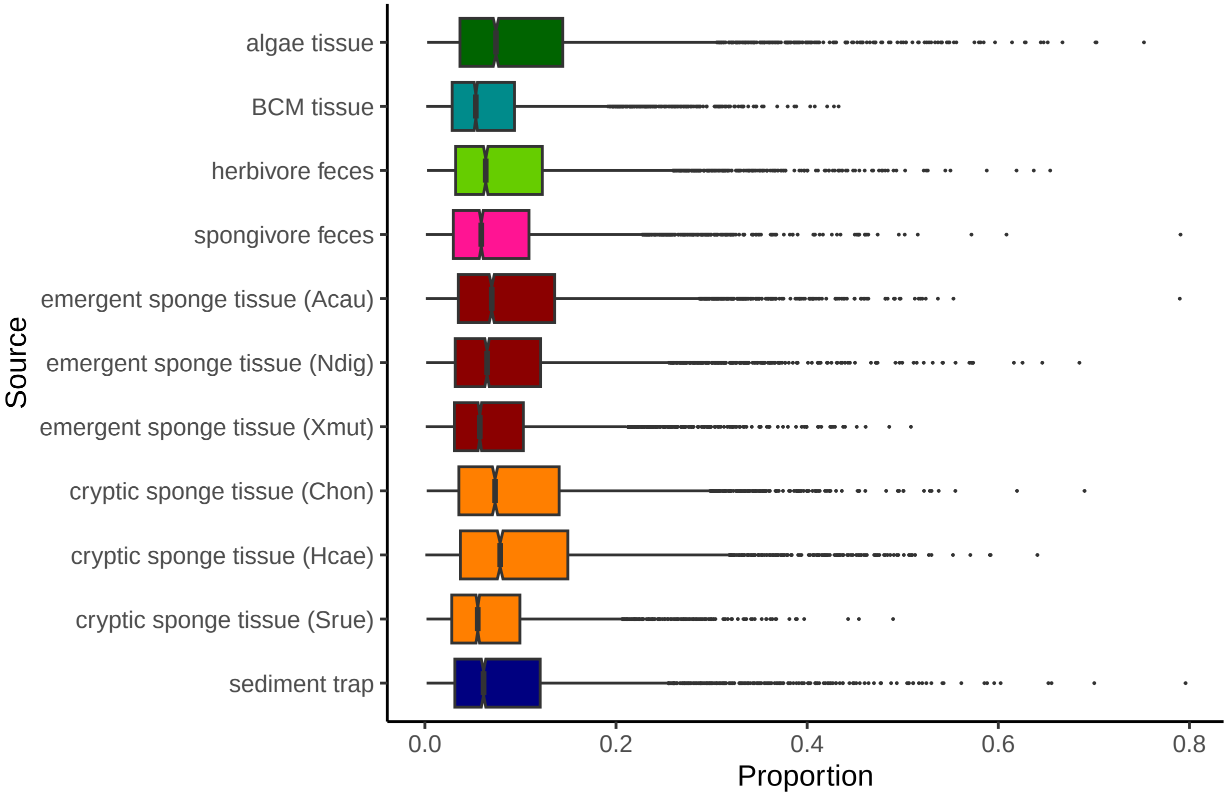
algae tissue
BCM tissue
herbivore feces
spongivore feces
emergent sponge tissue (Acau)
emergent sponge tissue (Ndig)
emergent sponge tissue (Xmut)
cryptic sponge tissue (Chon)
cryptic sponge tissue (Hcae)
cryptic sponge tissue (Srue)
sediment trap
Source
0.0
0.2
0.4
0.6
0.8
Proportion
